# Drug Repurposing for Alzheimer's Disease Based on Protein-Protein Interaction Network

**DOI:** 10.1155/2021/1280237

**Published:** 2021-10-14

**Authors:** Negar Sadat Soleimani Zakeri, Saeid Pashazadeh, Habib MotieGhader

**Affiliations:** ^1^Department of Computer Engineering, Faculty of Electrical and Computer Engineering, University of Tabriz, Tabriz, Iran; ^2^Department of Information Technology, Faculty of Electrical and Computer Engineering, University of Tabriz, Tabriz, Iran; ^3^Department of Computer Engineering, Gowgan Educational Center, Tabriz Branch, Islamic Azad University, Tabriz, Iran

## Abstract

Alzheimer's disease (AD) is known as a critical neurodegenerative disorder. It worsens as symptoms concerning dementia grow severe over the years. Due to the globalization of Alzheimer's disease, its prevention and treatment are vital. This study proposes a method to extract substantial gene complexes and then introduces potential drugs in Alzheimer's disease. To this end, a protein-protein interaction (PPI) network was utilized to extract five meaningful gene complexes functionally interconnected. An enrichment analysis to introduce the most important biological processes and pathways was accomplished on the obtained genes. The next step is extracting the drugs related to AD and introducing some new drugs which may be helpful for this disease. Finally, a complete network including all the genes associated with each gene complex group and genes' target drug was illustrated. For validating the proposed potential drugs, Connectivity Map (CMAP) analysis was accomplished to determine target genes that are up- or downregulated by proposed drugs. Medical studies and publications were analyzed thoroughly to introduce AD-related drugs. This analysis proves the accuracy of the proposed method in this study. Then, new drugs were introduced that can be experimentally examined as future work. Raloxifene and gentian violet are two new drugs, which have not been introduced as AD-related drugs in previous scientific and medical studies, recommended by the method of this study. Besides the primary goal, five bipartite networks representing the genes of each group and their target miRNAs were constructed to introduce target miRNAs.

## 1. Introduction

Alzheimer's disease, as a significant disease, has attracted researchers, especially in recent decades. Due to the importance of this disease, people should be informed to prevent the progression of the disease [1]. The recent developments and theoretic methods are introduced as a review study in this field [2]. Our previous study utilized a gene coexpression network to extract the biomarkers, including genes and miRNAs related to Alzheimer's disease [3]. This study develops a protein-protein interaction (PPI) network and a different methodology to introduce the associated biomarkers and drugs. Interactions between proteins are influential in cellular functions. Functionally related proteins are in identical complexes and organelles. Thus, uncovering this structure helps us recognize more about the role of genome variation in disease [4]. Studying the association of cellular functions and disease has received less attention, while it is as significant as studying the association of cellular functions and protein complexes [5]. Studying PPI networks helps us understand the nature of complicated diseases as it can highlight significant proteins which can be used in drug designing [6]. Many previous studies examined the relationship between disease and protein complexes. For example, in a paper with this context, the writer maintains that patients with some disorders, especially concerning the nervous system, have mutations in adaptor protein complexes [7]. Another paper uses the PPI network of AD and nonalcoholic fatty liver disease (NAFLD) to discover common pathways in these two diseases [6]. Also, analyzing PPI networks in AD and Parkinson's disease illustrates that similar proteins in the same clusters have analogous ontology and sequence different from other clusters [8]. Another related study combines gene network information with brain-tissue protein interactions and finds effective clusters of genes in AD and expression levels in this disease [9]. Many studies in computational biology are aimed at figuring out biomarkers in different diseases, especially in cancer. For instance, there is a study that introduces biomarkers in breast cancer [10] or another study that utilizes the PPI network to find the effective genes in AD progression [11]. Reviewing the previous studies indicates that protein complexes are not widely used to find effective genes and related drugs, especially in AD. Therefore, our research concentrates on these gaps and attempts to extract meaningful groups of genes. Materials and Methods introduces the database and the proposed method in this study. In Results, the dataset description, enrichment analysis of these gene groups, and the experiments' outputs are illustrated by relevant charts and tables. Discussion contains clinical and medical instances supporting the applicability of the proposed method.

## 2. Material and Methods

In this part, the used dataset is described in detail, and then, the adopted methodology and approaches are described.

### 2.1. Dataset

We utilized a dataset provided by Hu et al. by retrieving the studies related to the genetic association in Alzheimer's disease from PubMed. The writers of this paper have extensively reviewed 5298 reports. Then, by omitting the unrelated reports, 823 publications presenting more significant associations were chosen. Finally, there is a list of 431 genes known as Alzheimer-related genes [12]. In the following, a step-by-step method used in this study is described.

### 2.2. Gene Complex Extraction

We used the STRING database to construct a PPI network. This database provides a comprehensive global network that integrates all the available PPI information [13]. We just considered, in the process of constructing this network, the interactions that are obtained experimentally. One of the most important methods of extracting protein complexes is using cluster analysis in biological networks. ClusterViz is an application to analyze and visualize the clusters in biological networks and is supported by the Cytoscape platform. Cytoscape is a versatile platform and popularly used to visualize biological researches [14]. We used Cytoscape 3.7.0. in this study. ClusterViz uses three different clustering algorithms. We tested these three algorithms with different parameters and led to choose EAGLE to construct the gene complexes. The EAGLE algorithm (agglomerative hierarchical clustering based on maximal clique) is an agglomerative hierarchical clustering algorithm. In contrast with traditional agglomerative algorithms, EAGLE uses the set of maximal cliques more than the set of vertices [15]. There are also two other clustering algorithms named MCODE and FAG-EC.

### 2.3. Functional Enrichment Analysis

Gene ontology analysis and biological pathway analysis have been developed to accomplish enrichment analysis using the Database for Annotation, Visualization, and Integrated Discovery (DAVID) [16] that helped us extract biological mechanism and gene ontology information. The Kyoto Encyclopedia of Genes and Genomes (KEGG) database [17] was utilized to study the pathways they involve.

### 2.4. Drug-Gene Networks

In the next step, drug-gene networks were constructed using DGIdb, which represents drug-gene interactions from different resources. Its user-friendly interface facilitates searching and filtering for easy access. We used DGIdb 3.0 [18], which indicates a significant database update by updating resources and adding new resources.

### 2.5. Bipartite Gene-miRNA Networks

In this step, target miRNAs of the genes in each group were extracted separately using the miRWalk2.0. So, five bipartite gene-miRNA networks were constructed and illustrated by Cytoscape 3.7.0. The miRNAs, which have a more considerable degree, achieve a more regulatory role. So, subnetworks are constructed by selecting miRNAs with a more considerable degree and their related genes.

**Figure 1:**
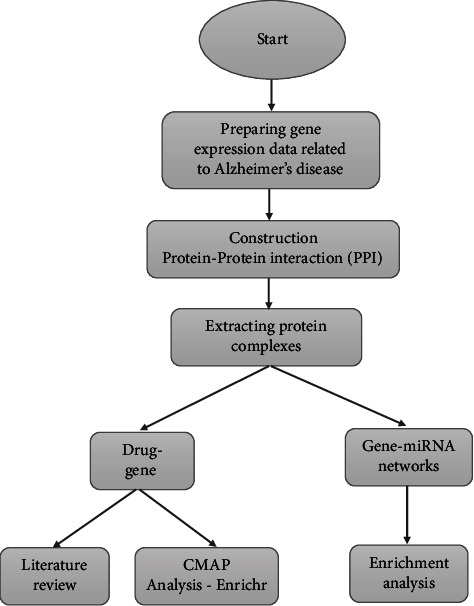
Workflow of the study.

In the summary of this section, the flow of the study is demonstrated by a flow chart diagram in Figure 1.

According to Figure 1, after preparing the dataset, the first step was constructing the PPI network and then extracting gene complexes. Enrichment analysis, including analysis on pathways and biological processes, was performed using the obtained gene complexes. Two types of bipartite networks, drug-gene networks and gene-miRNA networks, were constructed by having the gene complexes. According to the primary goal of this study, which is to introduce potential drugs, the drug-gene network was utilized to introduce significant drugs. For representing the target genes that the proposed drugs regulate, this part's results were analyzed using the CMAP database. Another analysis for the proposed drugs was reviewing previous experimental studies to prove the correctness of the methodology.

## 3. Results

This part describes the results extracted by different steps of the methodology.

### 3.1. Extracting Protein Complexes from Protein-Protein Interaction (PPI) Network

**Figure 2:**
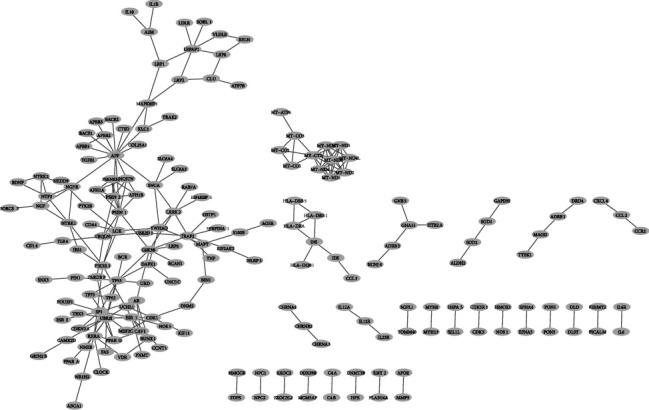
The PPI network with experimentally obtained interactions.

First, the STRING database [13], which includes all interactions between proteins, was utilized to construct a PPI network. The network that has been built for this study is a collection of genes with experimentally obtained interactions. The constructed PPI network is displayed in Figure 2.

**Figure 3:**
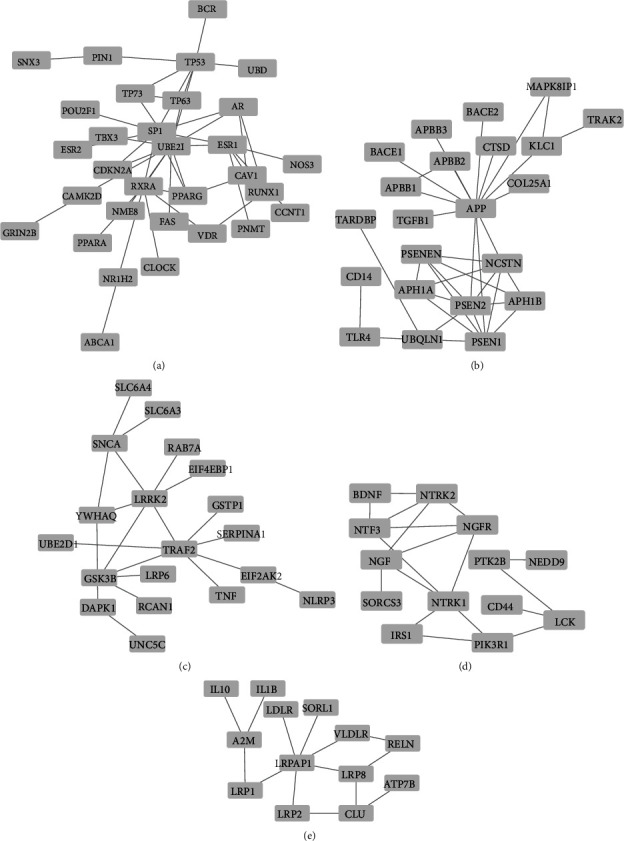
Obtained PPI clusters. The nodes represent each cluster genes: (a) cluster 1; (b) cluster 3; (c) cluster 6; (d) cluster 7; (e) cluster 8.

The extracted network is then clustered by the EAGLE algorithm, one of the algorithms in the ClusterViz application [14]. There are two parameters associated with the EAGLE algorithm, named “CliqueSize Threshold” and “ComplexSize.” After examining different values, 3 and 2 were selected as “CliqueSize Threshold” and “ComplexSize” parameters, respectively. Among the 9 clusters constructed by running this algorithm, 5 clusters were selected for the following research step. Four remaining clusters were eliminated because they were meaningless according to their structures and could not represent a gene complex. Five selected groups of genes are represented in Figure 3, and the list of genes for each cluster is provided in Table 1.

### 3.2. Enrichment Analysis of Genes

Functional enrichment analysis was accomplished for each cluster independently. The results are demonstrated in separate tables. The *p* value of the most significant pathway is equal to 4.16*E* − 04 and involved six genes. The related term to this pathway is transcriptional and is found in cluster 1. In cluster 3, the substantial pathway is Alzheimer's disease. Its *p* value is equal to 5.13*E* − 11, and it includes ten genes. In the sixth cluster, the most crucial pathway is hepatitis c. Its *p* value is equal to 0.004662, including four genes. In the next cluster, cluster 7, the most significant pathway is the neurotrophin signalling pathway with eight genes. Its *p* value is 4.74*E* − 11, and it includes eight important genes. In the last cluster, cluster 8, the most crucial pathway is malaria. Its *p* value is 7.32*E* − 04 and consists of three genes. These are the most significant pathways for each cluster. More comprehensive information, including all the pathways, is available in Additional File 1 (Supplementary Tables 3–7).

According to the gene ontology analysis, the most important biological processes for cluster 1 were, respectively, transcription from RNA polymerase II promoter (*p* value = 4.66*E* − 13), with 21 genes; regulation of transcription from RNA polymerase II promoter (*p* value = 5.45*E* − 13), with two genes; and positive regulation of cellular metabolic process (*p* value = 9.75*E* − 13) with 24 genes. For cluster 3, membrane protein proteolysis was the most significant biological process (*p* value = 1.78*E* − 15) and included nine genes. The next important processes were notch receptor processing (*p* value = 7.92*E* − 12) with six genes and membrane protein ectodomain proteolysis (*p* value = 8.90*E* − 12) with seven genes. In the sixth cluster, the most important biological process with 18 genes (*p* value = 3.91*E* − 09) was the negative regulation of the biological process. The next three important processes were, respectively, negative regulation of response to a stimulus with 12 genes (*p* value = 1.89*E* − 08), regulation of apoptotic process with 12 genes (*p* value = 2.39*E* − 08), and regulation of programmed cell death with 12 genes (*p* value = 2.64*E* − 08). In cluster 7, the important processes are, respectively, the transmembrane receptor protein tyrosine kinase signalling pathway with 11 genes (*p* value = 4.21*E* − 13), the enzyme-linked receptor protein signalling pathway with 11 genes (*p* value = 1.99*E* − 11), and cell surface receptor signalling pathway with 13 genes (*p* value = 2.55*E* − 10). In cluster 8, significant processes are, respectively, vesicle-mediated transport with 10 genes (*p* value = 5.86*E* − 08), endocytosis with 8 genes (*p* value = 9.86*E* − 08), transport with 13 genes (*p* value = 2.08*E* − 07), and establishment of localization with 13 genes (*p* value = 2.87*E* − 07). The other important biological processes sorted by their *p* value are available in separate tables in Additional File 1 (Supplementary Tables 8–12).

### 3.3. The Drug-Gene Interaction Network

**Figure 4:**
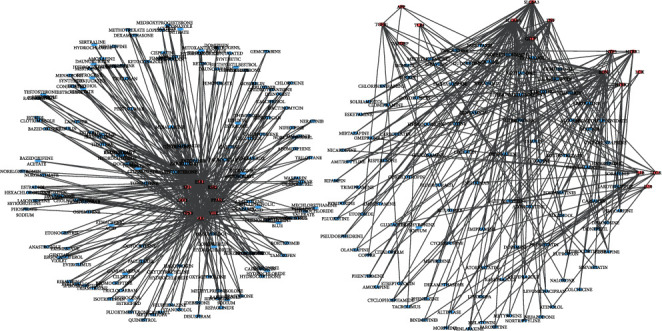
The drug-gene interaction network along with related protein complexes.

To introduce the potential drugs for Alzheimer's treatment, we used the DGIdb 3.0 database to extract drug-gene interactions [18]. By utilizing this database, medicines related to each cluster's genes are extracted and visualized in Figure 4.

The undirected associations between genes and drugs are illustrated. The relation between each gene group indicates the gene complexes, which are shown by separately drawn clusters. Blue hexagonal nodes and the red oval nodes represent the drugs and the genes. Medicines related to each cluster are illustrated in Additional File 1 (Supplementary Table 13).

### 3.4. Gene Set Enrichment Analysis (GSEA)

The Enrichr [19] database was utilized to perform CMAP analysis [20]. The central concept of this analysis is to evaluate the therapeutic effects of the proposed drugs. Two datasets called CMAP-up and CMAP-down, respectively, contain genes upregulated or downregulated by various extracted drugs. The results of this analysis, which represent their significance, are demonstrated in Table 2.

According to Table 2, nine of fourteen suggested drugs were found in the CMAP database. The first column shows drug names and the second one the degree of each drug. The third column demonstrates the target genes related to each drug. The last column shows regulating the status of genes according to the CMAP database if it is upregulated or downregulated by the related drug. The entries that are empty in the last column are the ones in which there is no regulating information in the CMAP database.

### 3.5. Bipartite miRNA-Gene Networks

**Figure 5:**
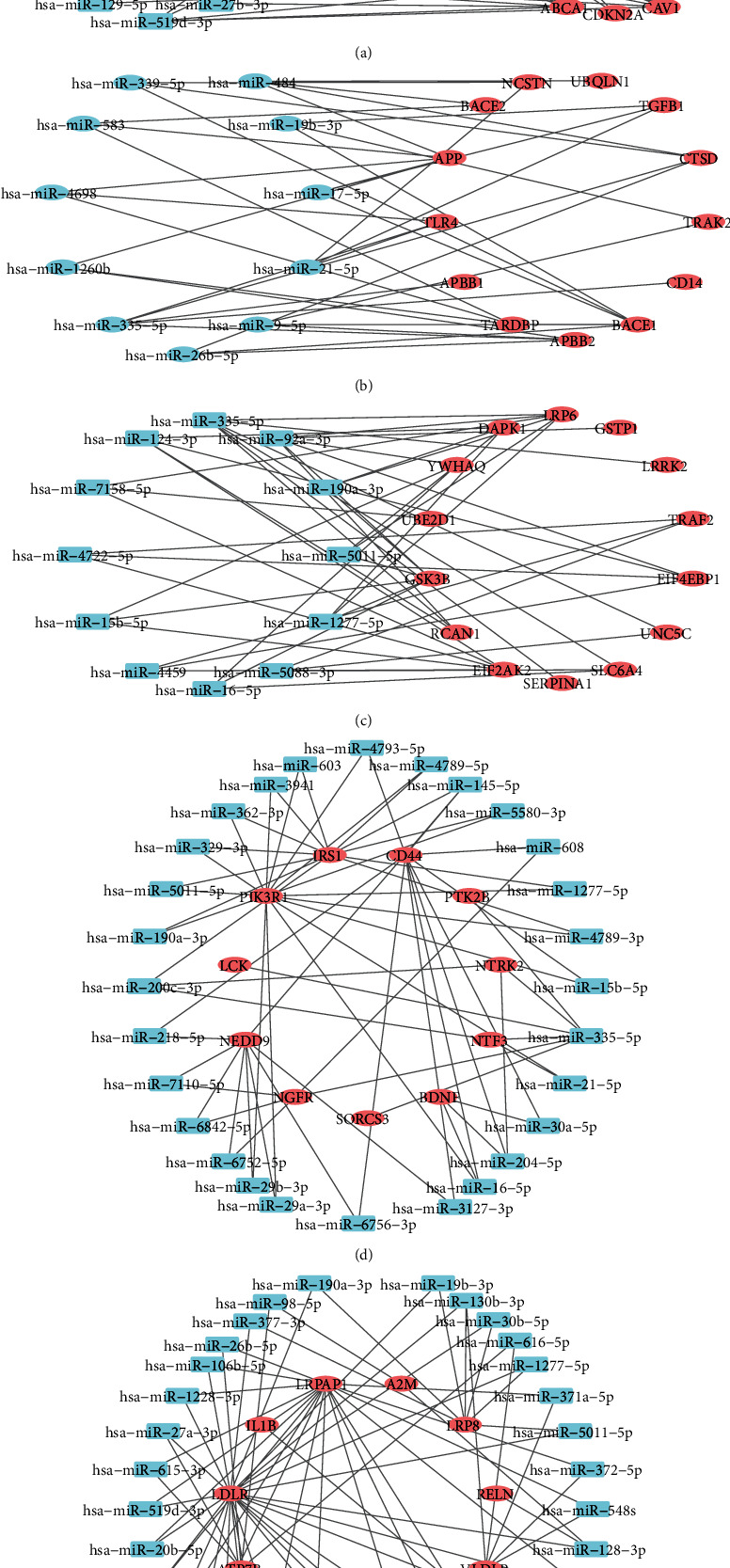
Bipartite gene-miRNA subnetworks for all of the clusters: (a) cluster 1; (b) cluster 3; (c) cluster 6; (d) cluster 7; (e) cluster 8.

Bipartite gene-miRNA networks, illustrated by Cytoscape 3.7.0, can examine complexes more in-depth. These bipartite networks are first analyzed by network specifications and then sorted by the degree of the nodes. The miRNAs with a higher degree and their associated genes and connections are normally selected.  Figure 5 shows bipartite gene-miRNA networks for each cluster. The red oval nodes represent the genes, and the blue rectangular ones signify their related miRNAs. There is a representation of the miRNAs concerning each cluster in Additional File 1 (Supplementary Table 1). After extracting bipartite networks, some of the genes are to be omitted. So, the lists of the genes are changed in the next step. These updated lists of genes are represented in Additional File 1 (Supplementary Table 2).

## 4. Discussion

In this article, we studied genes associated with Alzheimer's disease. Extracting PPI networks and then obtaining meaningful gene complexes led to finding efficient gene groups in Alzheimer's disease. Performing enrichment analysis over these gene groups, significant biological processes and pathways in the related part are listed. Target drugs of the selected gene groups were introduced and visualized explicitly having significant genes. In this way, each gene group was demonstrated by red oval nodes diagnosed as separate groups and blue hexagonal nodes as the common drugs related to different genes. In order to introduce effective miRNAs, target miRNAs were extracted, and bipartite subnetworks for each gene group were constructed for a secondary purpose. In the last step, to achieve the primary purpose of this study, the drugs are sorted by their degree value to launch a comprehensive inquiry of the obtained results. Since drugs with a higher degree are associated with more genes, fourteen drugs were selected; two of them tamoxifen and raloxifene were with a degree value of 7; haloperidol was with a degree of 6; doxorubicin, verapamil, gentian violet, and progesterone were with the degree of 5; and the rest with a degree value of 4, which are listed as the following: alteplase, pilocarpine, estradiol, hexachlorophene, daunorubicin hydrochloride, nicotine, and clozapine.

These are vital drugs introduced by this study for Alzheimer's disease. All 15 obtained drugs are reviewed in previous experimental and medical studies to acknowledge the accuracy of the present study's results.

Initially, tamoxifen and verapamil were examined, reviewing the previous studies. A case-control study in Taiwan examined the relation between tamoxifen use and AD and concluded that using this drug for a long time tends to the longer life of Alzheimer's patients [21]. Another study indicated the role of tamoxifen in Alzheimer's disease and found that it enhances memory [22]. Another study showed that tamoxifen contributes to reducing impairment and improvement of the learning system [23].

According to the literature, a study introduced verapamil as a medication for male and female samples dealing with Alzheimer's disease [24]. According to a review study, verapamil, previously used for cardiovascular disease, is currently considered a treatment for neurological disorders [25]. In addition to Alzheimer's disease, verapamil is reported as a treatment in Huntington and Parkinson's diseases [26, 27].

The following thirteen drugs were examined as follows. Alteplase, also known as t-PA, contributes to reducing AD-related pathology by making progress in the cognitive function of the sample [28]. Another study about this treatment method indicated that alteplase prevents ischemic brain lesions from being progressed while imaging after the stroke [29]. Another published paper maintained the role of t-PA in omitting A*β* (deposition of the beta-amyloid peptide as one of the important reasons for Alzheimer's disease). This study also indicated that t-PA reduces the speed of Alzheimer's disease progression [30]. The next one is pilocarpine which is claimed to be instructive in detecting patients with AD and dementia [31]. However, there is no other related study. Raloxifene, the next one, which has a neuroprotective role [32], was maintained to help decrease inflammation of the brain. Another study claimed that raloxifene prevents cell death in neurons and meliorates mild cognitive impairment in examined samples. Its beneficial effects on Parkinson's disease [33] were maintained as well. Another study concluded that 120 mg per day as the dose of raloxifene reduces cognitive impairment risk [34]. The next one is gentian violet, yet there is no study on the association of gentian violet and Alzheimer's disease. The next one, hexachlorophene, is almost associated with neurodegenerative disease and helps reduction of A*β*_42_-induced toxicity and prevents neural damage [35]. Another study indicated hexachlorophene as a potential drug in Alzheimer's disease treatment by regulating tau levels [36]. Nicotine affects the aetiology of Alzheimer's disease and Parkinson's disease; however, the writers did not recommend it for its other health problems [37]. Another paper observed cognitive progress using nicotine in mild cognitive impairment (MCI) samples [38]. Doxorubicin represents a protective effect on the brain over chemotherapy-induced impairment (CICI) [39]. The subsequent study investigated their relationship with cognitive abilities and introduced more evidence about its effect [40]. There is no direct relation between AD and this drug, so this study proposes it as an Alzheimer-related drug to be studied extensively. The next drug is haloperidol that has an association with patients with Alzheimer's disease who have behavior problems and represents relatively good changes in patients' behavior [41]. The next study indicated that using a special dose of haloperidol decreases behavioral problems (psychosis and destructive behaviors) in the abovementioned patients [42]. Another study showed a decrement in delays for finding new objects through the examinations [43]. Almost all the studies related to this drug, reviewed in this part, are not directly related to AD, but they are indirectly associated with changes in behaviors of patients with Alzheimer's disease or dementia. The next drug is daunorubicin hydrochloride which is known as daunomycin, with trade names Adriamycin, Cerubidine, and Blenoxane [44]. A study stated that the compounds containing daunomycin prevent Abeta fibril formation and slow down the progression of Alzheimer's disease [45]. This is the only study that noted the relation between Alzheimer's disease and daunomycin. Another study proposed it for the long-term treatment of patients with Alzheimer's disease. Its results also maintained its effect in the reduction of amyloid-beta (A*β*) deposition [46]. Another study also introduced clozapine for psychosis treatment in patients affected by Alzheimer's dementia or Parkinson's disease [47]. The next paper introduced clozapine as the related drug for Alzheimer's treatment that improves short-term memory [48]. The next drug is estradiol which is stated as a useful drug for the enhancement of cognitive functions [49]. The other study maintained that estradiol in higher levels provides a higher covariance in-memory network and better cognitive health specially for women [50]. There are two other studies that suggested using estradiol as a therapy in Alzheimer's disease [51, 52]. The last one is progesterone which contributes to progression in learning and memory abilities by improving glucose metabolism in neurons [53]. Another article maintained that progesterone increases cognitive abilities, prevents A*β* inflammation, and is a therapy method in Alzheimer's disease [54]. The next study asserted some behavioral problems like depression caused by Alzheimer's disease and claimed that progesterone decreases these behavioral problems [55].

According to the extensive and detailed review, the drugs extracted by this article can be divided into three groups. The first group is about AD-related drugs introduced by previously published researches. The second group is the drugs that are not directly related to AD, or there has not been enough study about them so far. The third group is about the medicines that have not been mentioned concerning AD in previous studies. This categorization is presented in Table 3.

The first group's drugs prove the accuracy of the proposed method in this study because the obtained results are consistent with previous medical publications. The second list of drugs is proposed earlier, but they can be studied more in detail as future works. The two drugs in the third group are suggested by this study as Alzheimer-related drugs, which can be examined experimentally.

## 5. Conclusions

The presented study is aimed at suggesting Alzheimer's disease-related drugs and introducing significant processes and pathways by extracting important gene complexes. The proposed method utilized PPI networks to extract these gene complexes. Then, for each gene complex, a bipartite subnetwork was drawn using their most important target miRNAs. In the next phase of the study, drug-gene networks were extracted and illustrated, along with related gene groups. By detailed investigation over the selected drugs, gentian violet was proposed as Alzheimer-related drugs by this study. The significance of the findings in this study was suggesting potential candidate drugs related to Alzheimer's disease. The candidate drugs can be experimentally investigated in future works.

## Figures and Tables

**Table 1:** List of the selected clusters along with their genes.

Number of clusters	Number of nodes	Nodes
1	31	PPARG, CAMK2D, RXRA, SP1, ESR2, GRIN2B, FAS, POU2F1, CDKN2A, NOS3, AR, CCNT1, ABCA1, RUNX1, VDR, NME8, UBE2I, TP63, TP73, PPARA, NR1H2, UBD, PNMT, TBX3, ESR1, CLOCK, SNX3, PIN1, TP53, BCR, CAV1
3	22	PSENEN, APH1A, TARDBP, NCSTN, PSEN1, APP, UBQLN1, APBB1, APBB3, COL25A1, BACE2, BACE1, TRAK2, KLC1, CTSD, MAPK8IP1, APH1B, TGFB1, CD14, APBB2, PSEN2, TLR4
6	19	GSTP1, DAPK1, EIF2AK2, SLC6A3, SNCA, UBE2D1, LRP6, NLRP3, RAB7A, LRRK2, YWHAQ, GSK3B, TRAF2, TNF, SLC6A4, RCAN1, EIF4EBP1, UNC5C, SERPINA1
7	13	NEDD9, NGF, SORCS3, NTRK1, LCK, NTRK2, PTK2B, BDNF, NGFR, NTF3, IRS1, PIK3R1, CD44
8	13	RELN, IL10, IL1B, LDLR, VLDLR, SORL1, LRP8, LRPAP1, LRP2, LRP1, A2M, ATP7B, CLU

**Table 2:** The CMAP analysis on potential candidate drugs.

Drug	Degree	Genes	Up- or downregulated genes
Tamoxifen	7	TGFB1, NTRK1, AR, TP53, ESR1, ESR2, VDR	TP53-up
Raloxifene	7	IL1B, TP53, ESR1, ESR2, VDR, AR, PPARG	AR-up
Haloperidol	6	SLC6A3, SLC6A4, BDNF, LDLR, AR, TP53	LDLR-up TP53-up
Doxorubicin	5	TGFB1, GSTP1, NTRK2, BDNF	TGFB1-up
Verapamil	5	TGFB1, SLC6A4, GSTP1, LDLR, IL1B	—
Progesterone	5	TARDBP, AR, TP53, ESR1, ESR2	TP53-up
Clozapine	4	SLC6A3, SLC6A4, GSTP1, NTRK2	GSTP1-down
Estradiol	4	AR, ESR1, ESR2, PPARG	AR-up ESR1-up ESR2-up
Pilocarpine	4	NTRK2, NTRK1, NTF3, BDNF	—

**Table 3:** A categorization of the extracted drugs.

Number of drugs	Drug names
9	Tamoxifen, verapamil, alteplase, raloxifene, hexachlorophene, nicotine, clozapine, estradiol, progesterone
4	Pilocarpine, doxorubicin, haloperidol, daunomycin
1	Gentian violet

## Data Availability

The results of analysis in terms of tables used to support the findings of this study are included within the supplementary information file.

## Supplementary Materials

Supplementary Materials Additional tables are represented in a supplementary file. This word file is called “supplementary”.
