# Supplementary material for: Drug Repurposing for Alzheimer's Disease Based on Protein-Protein Interaction Network
Source: Biomed Res Int. 2021 Oct 14;2021:1280237. doi: 10.1155/2021/1280237 (PMC8531773; doi:10.1155/2021/1280237)
Supplement: Supplementary Materials — Additional tables are represented in a supplementary file. This word file is called “supplementary”. [file 1280237.f1.docx]

**Drug Repurposing for Alzheimer’s Disease Based on Protein-Protein Interaction Network**

Negar Sadat Soleimani Zakeri^1^, Saeid Pashazadeh^2^, Habib MotieGhader^3^

1. Department of Computer Engineering, Faculty of Electrical and Computer Engineering, University of Tabriz, Tabriz, Iran.
2. Department of Information Technology, Faculty of Electrical and Computer Engineering, University of Tabriz, Tabriz, Iran.

3. Department of Computer Engineering, Gowgan Educational Center, Tabriz Branch, Islamic Azad University, Tabriz, Iran.

**Table S1.** The list of miRNAs related to each cluster

| Cluster | Number of miRNAs | List of miRNAs |
| --- | --- | --- |
| Cluster1 | 19 | hsa-miR-27b-3p, hsa-miR-19b-3p, hsa-miR-19a-3p, hsa-miR-17-5p, hsa-miR-106b-5p, hsa-miR-21-5p, hsa-miR-106a-5p, hsa-miR-34a-5p, hsa-miR-190a-3p, hsa-miR-124-3p, hsa-miR-20a-5p, hsa-miR-20b-5p, hsa-miR-27a-3p, hsa-miR-6749-3p, hsa-miR-335-5p, hsa-miR-26b-5p, hsa-miR-129-5p, hsa-miR-519d-3p, hsa-miR-128-3p |
| Cluster3 | 11 | hsa-miR-339-5p, hsa-miR-484, hsa-miR-335-5p, hsa-miR-26b-5p, hsa-miR-19b-3p, hsa-miR-9-5p, hsa-miR-17-5p, hsa-miR-1260b, hsa-miR-4698, hsa-miR-583, hsa-miR-21-5p |
| Cluster6 | 12 | hsa-miR-1277-5p, hsa-miR-5011-5p, hsa-miR-15b-5p, hsa-miR-190a-3p, hsa-miR-92a-3p, hsa-miR-16-5p, hsa-miR-7158-5p, hsa-miR-124-3p, hsa-miR-335-5p, hsa-miR-5088-3p, hsa-miR-4722-5p, hsa-miR-4459 |
| Cluster7 | 28 | hsa-miR-6752-5p, hsa-miR-335-5p, hsa-miR-6842-5p, hsa-miR-7110-5p, hsa-miR-608, hsa-miR-29b-3p, hsa-miR-16-5p, hsa-miR-603, hsa-miR-21-5p, hsa-miR-3941, hsa-miR-4789-3p, hsa-miR-5011-5p, hsa-miR-5580-3p, hsa-miR-190a-3p, hsa-miR-4789-5p, hsa-miR-4793-5p, hsa-miR-29a-3p, hsa-miR-15b-5p, hsa-miR-362-3p, hsa-miR-1277-5p, hsa-miR-329-3p, hsa-miR-218-5p, hsa-miR-3127-3p, hsa-miR-30a-5p, hsa-miR-6756-3p, hsa-miR-204-5p, hsa-miR-145-5p, hsa-miR-200c-3p |
| Cluster8 | 35 | hsa-miR-98-5p, hsa-miR-27a-3p, hsa-miR-27b-3p, hsa-miR-615-3p, hsa-miR-616-5p, hsa-miR-26b-5p, hsa-miR-106a-5p, hsa-miR-106b-5p, hsa-miR-17-5p, hsa-miR-20a-5p, hsa-miR-1228-3p, hsa-miR-20b-5p, hsa-miR-548s, hsa-miR-519d-3p, hsa-miR-526b-3p, hsa-miR-93-5p, hsa-miR-519a-3p, hsa-miR-519b-3p, hsa-miR-371a-5p, hsa-miR-519c-3p, hsa-miR-372-5p, hsa-miR-4668-3p, hsa-miR-128-3p, hsa-miR-338-5p, hsa-miR-520h, hsa-miR-520g-3p, hsa-miR-335-5p, hsa-miR-130b-3p, hsa-miR-1277-5p, hsa-miR-377-3p, hsa-miR-30b-5p, hsa-miR-190a-3p, hsa-miR-5011-5p, hsa-miR-19b-3p, hsa-miR-21-5p |

**Table S2.** List of genes related to each gene group.

| **Cluster Name** | **Number of Genes** | **List of Genes** |
| --- | --- | --- |
| Cluster1 | 24 | CCNT1, FAS, AR, PPARG, UBD, CLOCK, TP53, NOS3, VDR, PPARA, ESR1, TBX3, RXRA, ESR2, SP1, ABCA1, CDKN2A, CAV1, SNX3, TP63, GRIN2B, POU2F1, PNMT, RUNX1 |
| Cluster3 | 13 | CTSD, TRAK2, CD14, BACE1, APBB2, TARDBP, APBB1, TLR4, APP, BACE2, NCSTN, UBQLN1, TGFB1 |
| Cluster6 | 14 | GSK3B, UBE2D1, YWHAQ, DAPK1, LRP6, GSTP1, LRRK2, TRAF2, EIF4EBP1, UNC5C, SLC6A4, SERPINA1, EIF2AK2, RCAN1 |
| Cluster7 | 11 | NGFR, PIK3R1, CD44, NTRK2, NTF3, BDNF, SORCS3, NEDD9, LCK, IRS1, PTK2B |
| Cluster8 | 11 | ATP7B, LRPAP1, A2M, VLDLR, LDLR, IL1B, LRP8, RELN, IL10, CLU, LRP1 |

**Table S3.** Pathways related to the first group of genes (Cluster1)

| **Term** | **Count** | **PValue** | **Genes** |
| --- | --- | --- | --- |
| Transcriptional misregulation in cancer | 6 | 4.16E-04 | RXRA, CCNT1, SP1, PPARG, TP53, RUNX1 |
| Pathways in cancer | 8 | 6.16E-04 | BCR, AR, RXRA, CDKN2A, FAS, PPARG, TP53, RUNX1 |
| p53 signaling pathway | 4 | 0.002186 | CDKN2A, FAS, TP53, TP73 |
| Chronic myeloid leukemia | 4 | 0.002686 | BCR, CDKN2A, TP53, RUNX1 |
| Thyroid cancer | 3 | 0.005643 | RXRA, PPARG, TP53 |
| Estrogen signaling pathway | 4 | 0.006583 | SP1, NOS3, ESR1, ESR2 |
| Proteoglycans in cancer | 5 | 0.007201 | CAMK2D, CAV1, FAS, ESR1, TP53 |
| Non-small cell lung cancer | 3 | 0.020057 | RXRA, CDKN2A, TP53 |
| Glioma | 3 | 0.026512 | CAMK2D, CDKN2A, TP53 |
| PPAR signaling pathway | 3 | 0.028047 | RXRA, PPARG, PPARA |
| Huntington's disease | 4 | 0.038291 | SP1, PPARG, TP53, GRIN2B |
| Insulin resistance | 3 | 0.06647 | NOS3, NR1H2, PPARA |
| Thyroid hormone signaling pathway | 3 | 0.074167 | RXRA, ESR1, TP53 |
| Neurotrophin signaling pathway | 3 | 0.079836 | CAMK2D, TP53, TP73 |
| Dopaminergic synapse | 3 | 0.08918 | CAMK2D, CLOCK, GRIN2B |
| Hepatitis C | 3 | 0.095182 | RXRA, PPARA, TP53 |
| Measles | 3 | 0.095182 | FAS, TP53, TP73 |
| MicroRNAs in cancer | 4 | 0.0999 | UBE2I, CDKN2A, TP53, TP63 |

**Table S4.** Pathways related to the third group of genes (Cluster3)

| **Term** | **Count** | **PValue** | **Genes** |
| --- | --- | --- | --- |
| Alzheimer's disease | 10 | 5.13E-11 | APH1A, BACE1, PSENEN, BACE2, APP, NCSTN, APH1B, PSEN2, APBB1, PSEN1 |
| Notch signaling pathway | 6 | 7.76E-08 | APH1A, PSENEN, NCSTN, APH1B, PSEN2, PSEN1 |
| Tuberculosis | 4 | 0.008731 | TGFB1, CD14, CTSD, TLR4 |
| Salmonella infection | 3 | 0.017394 | CD14, KLC1, TLR4 |
| Amoebiasis | 3 | 0.027516 | TGFB1, CD14, TLR4 |

**Table S5.** Pathways related to the sixth group of genes (Cluster6)

| **Term** | **Count** | **PValue** | **Genes** |
| --- | --- | --- | --- |
| Hepatitis C | 4 | 0.004662 | GSK3B, EIF2AK2, TRAF2, TNF |
| Influenza A | 4 | 0.009813 | GSK3B, NLRP3, EIF2AK2, TNF |
| Parkinson's disease | 3 | 0.052171 | LRRK2, SLC6A3, SNCA |
| Non-alcoholic fatty liver disease (NAFLD) | 3 | 0.058212 | GSK3B, TRAF2, TNF |
| Alzheimer's disease | 3 | 0.070255 | GSK3B, TNF, SNCA |
| Protein processing in endoplasmic reticulum | 3 | 0.070988 | UBE2D1, EIF2AK2, TRAF2 |
| Pathways in cancer | 4 | 0.0798 | GSK3B, DAPK1, GSTP1, TRAF2 |
| Herpes simplex infection | 3 | 0.081514 | EIF2AK2, TRAF2, TNF |
| Viral carcinogenesis | 3 | 0.098979 | YWHAQ, EIF2AK2, TRAF2 |

**Table S6.** Pathways related to the seventh group of genes (Cluster7)

| **Term** | **Count** | **PValue** | **Genes** |
| --- | --- | --- | --- |
| Neurotrophin signaling pathway | 8 | 4.74E-11 | NTRK1, NTRK2, NGFR, BDNF, IRS1, NTF3, PIK3R1, NGF |
| MAPK signaling pathway | 5 | 3.15E-04 | NTRK1, NTRK2, BDNF, NTF3, NGF |
| Apoptosis | 3 | 0.003433 | NTRK1, PIK3R1, NGF |
| Inflammatory mediator regulation of TRP channels | 3 | 0.008392 | NTRK1, PIK3R1, NGF |
| PI3K-Akt signaling pathway | 4 | 0.011529 | NGFR, IRS1, PIK3R1, NGF |
| Natural killer cell mediated cytotoxicity | 3 | 0.012791 | LCK, PTK2B, PIK3R1 |
| Rap1 signaling pathway | 3 | 0.035517 | NGFR, PIK3R1, NGF |
| Ras signaling pathway | 3 | 0.040641 | NGFR, PIK3R1, NGF |
| Aldosterone-regulated sodium reabsorption | 2 | 0.055305 | IRS1, PIK3R1 |
| Type II diabetes mellitus | 2 | 0.06767 | IRS1, PIK3R1 |
| Regulation of lipolysis in adipocytes | 2 | 0.078538 | IRS1, PIK3R1 |
| mTOR signaling pathway | 2 | 0.081238 | IRS1, PIK3R1 |
| Central carbon metabolism in cancer | 2 | 0.089293 | NTRK1, PIK3R1 |

**Table S7.** Pathways related to the eighth group of genes (Cluster8)

| **Term** | **Count** | **PValue** | **Genes** |
| --- | --- | --- | --- |
| Malaria | 3 | 7.32E-04 | IL10, LRP1, IL1B |
| African trypanosomiasis | 2 | 0.02845 | IL10, IL1B |
| Inflammatory bowel disease (IBD) | 2 | 0.054559 | IL10, IL1B |
| Leishmaniasis | 2 | 0.060373 | IL10, IL1B |
| Pertussis | 2 | 0.063682 | IL10, IL1B |
| Chagas disease (American trypanosomiasis) | 2 | 0.087381 | IL10, IL1B |
| Amoebiasis | 2 | 0.088997 | IL10, IL1B |
| Toxoplasmosis | 2 | 0.092222 | IL10, LDLR |

**Table S8.** Biological processes related to the first group of genes (Cluster 1)

| **Term** | **Count** | **PValue** | **Genes** |
| --- | --- | --- | --- |
| GO:0006366~transcription from RNA polymerase II promoter | 21 | 4.66E-13 | UBE2I, POU2F1, CCNT1, CDKN2A, VDR, NR1H2, CAV1, ESR1, TBX3, ESR2, RUNX1, AR, RXRA, SP1, PIN1, PPARG, PPARA, CLOCK, TP53, TP63, TP73 |
| GO:0006357~regulation of transcription from RNA polymerase II promoter | 21 | 5.45E-13 | CAMK2D, UBE2I, CCNT1, CDKN2A, VDR, NR1H2, CAV1, ESR1, TBX3, ESR2, RUNX1, AR, RXRA, SP1, PIN1, PPARG, PPARA, CLOCK, TP53, TP63, TP73 |
| GO:0031325~positive regulation of cellular metabolic process | 24 | 9.75E-13 | ABCA1, CAMK2D, UBE2I, CCNT1, CDKN2A, NOS3, VDR, NR1H2, CAV1, ESR1, TBX3, ESR2, RUNX1, AR, RXRA, SP1, FAS, PIN1, PPARG, PPARA, CLOCK, TP53, TP63, TP73 |
| GO:0045935~positive regulation of nucleobase-containing compound metabolic process | 20 | 1.34E-12 | ABCA1, CCNT1, CDKN2A, NOS3, VDR, NR1H2, ESR1, TBX3, ESR2, RUNX1, AR, RXRA, SP1, PIN1, PPARG, PPARA, CLOCK, TP53, TP63, TP73 |
| GO:0051253~negative regulation of RNA metabolic process | 18 | 2.47E-12 | UBE2I, POU2F1, CCNT1, CDKN2A, VDR, NR1H2, CAV1, ESR1, TBX3, ESR2, RUNX1, RXRA, PPARG, PPARA, CLOCK, TP53, TP63, TP73 |
| GO:0031328~positive regulation of cellular biosynthetic process | 20 | 2.84E-12 | ABCA1, CCNT1, CDKN2A, NOS3, VDR, NR1H2, ESR1, TBX3, ESR2, RUNX1, AR, RXRA, SP1, PIN1, PPARG, PPARA, CLOCK, TP53, TP63, TP73 |
| GO:0048523~negative regulation of cellular process | 27 | 3.48E-12 | CAMK2D, CCNT1, SNX3, RXRA, TP63, ABCA1, POU2F1, UBE2I, CDKN2A, NOS3, VDR, NR1H2, CAV1, ESR1, TBX3, ESR2, RUNX1, BCR, AR, NME8, FAS, PIN1, PPARG, PPARA, CLOCK, TP53, TP73 |
| GO:0009891~positive regulation of biosynthetic process | 20 | 3.91E-12 | ABCA1, CCNT1, CDKN2A, NOS3, VDR, NR1H2, ESR1, TBX3, ESR2, RUNX1, AR, RXRA, SP1, PIN1, PPARG, PPARA, CLOCK, TP53, TP63, TP73 |
| GO:0051173~positive regulation of nitrogen compound metabolic process | 20 | 4.20E-12 | ABCA1, CCNT1, CDKN2A, NOS3, VDR, NR1H2, ESR1, TBX3, ESR2, RUNX1, AR, RXRA, SP1, PIN1, PPARG, PPARA, CLOCK, TP53, TP63, TP73 |
| GO:0009893~positive regulation of metabolic process | 24 | 4.68E-12 | ABCA1, CAMK2D, UBE2I, CCNT1, CDKN2A, NOS3, VDR, NR1H2, CAV1, ESR1, TBX3, ESR2, RUNX1, AR, RXRA, SP1, FAS, PIN1, PPARG, PPARA, CLOCK, TP53, TP63, TP73 |
| GO:0045892~negative regulation of transcription, DNA-templated | 17 | 9.44E-12 | UBE2I, POU2F1, CDKN2A, VDR, NR1H2, CAV1, ESR1, TBX3, ESR2, RUNX1, RXRA, PPARG, PPARA, CLOCK, TP53, TP63, TP73 |
| GO:0045893~positive regulation of transcription, DNA-templated | 18 | 9.59E-12 | CCNT1, CDKN2A, VDR, NR1H2, ESR1, TBX3, ESR2, RUNX1, AR, RXRA, SP1, PIN1, PPARG, PPARA, CLOCK, TP53, TP63, TP73 |
| GO:1903508~positive regulation of nucleic acid-templated transcription | 18 | 9.59E-12 | CCNT1, CDKN2A, VDR, NR1H2, ESR1, TBX3, ESR2, RUNX1, AR, RXRA, SP1, PIN1, PPARG, PPARA, CLOCK, TP53, TP63, TP73 |
| GO:1902680~positive regulation of RNA biosynthetic process | 18 | 1.21E-11 | CCNT1, CDKN2A, VDR, NR1H2, ESR1, TBX3, ESR2, RUNX1, AR, RXRA, SP1, PIN1, PPARG, PPARA, CLOCK, TP53, TP63, TP73 |
| GO:0045934~negative regulation of nucleobase-containing compound metabolic process | 18 | 1.24E-11 | UBE2I, POU2F1, CCNT1, CDKN2A, VDR, NR1H2, CAV1, ESR1, TBX3, ESR2, RUNX1, RXRA, PPARG, PPARA, CLOCK, TP53, TP63, TP73 |
| GO:1903507~negative regulation of nucleic acid-templated transcription | 17 | 1.75E-11 | UBE2I, POU2F1, CDKN2A, VDR, NR1H2, CAV1, ESR1, TBX3, ESR2, RUNX1, RXRA, PPARG, PPARA, CLOCK, TP53, TP63, TP73 |
| GO:0051254~positive regulation of RNA metabolic process | 18 | 2.06E-11 | CCNT1, CDKN2A, VDR, NR1H2, ESR1, TBX3, ESR2, RUNX1, AR, RXRA, SP1, PIN1, PPARG, PPARA, CLOCK, TP53, TP63, TP73 |
| GO:0048519~negative regulation of biological process | 27 | 2.08E-11 | CAMK2D, CCNT1, SNX3, RXRA, TP63, ABCA1, POU2F1, UBE2I, CDKN2A, NOS3, VDR, NR1H2, CAV1, ESR1, TBX3, ESR2, RUNX1, BCR, AR, NME8, FAS, PIN1, PPARG, PPARA, CLOCK, TP53, TP73 |
| GO:0010558~negative regulation of macromolecule biosynthetic process | 18 | 2.13E-11 | UBE2I, POU2F1, CDKN2A, VDR, NR1H2, CAV1, ESR1, TBX3, ESR2, RUNX1, AR, RXRA, PPARG, PPARA, CLOCK, TP53, TP63, TP73 |
| GO:1902679~negative regulation of RNA biosynthetic process | 17 | 2.17E-11 | UBE2I, POU2F1, CDKN2A, VDR, NR1H2, CAV1, ESR1, TBX3, ESR2, RUNX1, RXRA, PPARG, PPARA, CLOCK, TP53, TP63, TP73 |
| GO:0010628~positive regulation of gene expression | 19 | 2.78E-11 | CCNT1, CDKN2A, VDR, NR1H2, CAV1, ESR1, TBX3, ESR2, RUNX1, AR, RXRA, SP1, PIN1, PPARG, PPARA, CLOCK, TP53, TP63, TP73 |
| GO:0048522~positive regulation of cellular process | 27 | 3.93E-11 | CAMK2D, CCNT1, SNX3, RXRA, UBD, TP63, ABCA1, UBE2I, CDKN2A, NOS3, VDR, NR1H2, CAV1, ESR1, TBX3, ESR2, RUNX1, BCR, AR, SP1, FAS, PIN1, PPARG, PPARA, CLOCK, TP53, TP73 |
| GO:0010629~negative regulation of gene expression | 18 | 4.45E-11 | UBE2I, POU2F1, CCNT1, CDKN2A, VDR, NR1H2, CAV1, ESR1, TBX3, ESR2, RUNX1, RXRA, PPARG, PPARA, CLOCK, TP53, TP63, TP73 |
| GO:0045944~positive regulation of transcription from RNA polymerase II promoter | 16 | 4.47E-11 | CCNT1, CDKN2A, VDR, NR1H2, ESR1, RUNX1, AR, RXRA, SP1, PIN1, PPARG, PPARA, CLOCK, TP53, TP63, TP73 |
| GO:0010605~negative regulation of macromolecule metabolic process | 21 | 4.66E-11 | UBE2I, POU2F1, CCNT1, CDKN2A, VDR, NR1H2, CAV1, ESR1, TBX3, ESR2, RUNX1, SNX3, AR, RXRA, PIN1, PPARG, PPARA, CLOCK, TP53, TP63, TP73 |
| GO:0009890~negative regulation of biosynthetic process | 18 | 5.44E-11 | UBE2I, POU2F1, CDKN2A, VDR, NR1H2, CAV1, ESR1, TBX3, ESR2, RUNX1, AR, RXRA, PPARG, PPARA, CLOCK, TP53, TP63, TP73 |
| GO:0051172~negative regulation of nitrogen compound metabolic process | 18 | 5.56E-11 | UBE2I, POU2F1, CCNT1, CDKN2A, VDR, NR1H2, CAV1, ESR1, TBX3, ESR2, RUNX1, RXRA, PPARG, PPARA, CLOCK, TP53, TP63, TP73 |
| GO:2000113~negative regulation of cellular macromolecule biosynthetic process | 17 | 1.03E-10 | UBE2I, POU2F1, CDKN2A, VDR, NR1H2, CAV1, ESR1, TBX3, ESR2, RUNX1, RXRA, PPARG, PPARA, CLOCK, TP53, TP63, TP73 |
| GO:0019438~aromatic compound biosynthetic process | 26 | 1.05E-10 | PNMT, CAMK2D, CCNT1, RXRA, TP63, ABCA1, POU2F1, UBE2I, CDKN2A, NOS3, VDR, NR1H2, CAV1, ESR1, TBX3, ESR2, RUNX1, AR, SP1, NME8, PIN1, PPARG, PPARA, CLOCK, TP53, TP73 |
| GO:0010557~positive regulation of macromolecule biosynthetic process | 18 | 1.41E-10 | CCNT1, CDKN2A, VDR, NR1H2, ESR1, TBX3, ESR2, RUNX1, AR, RXRA, SP1, PIN1, PPARG, PPARA, CLOCK, TP53, TP63, TP73 |
| GO:1901362~organic cyclic compound biosynthetic process | 26 | 1.86E-10 | PNMT, CAMK2D, CCNT1, RXRA, TP63, ABCA1, POU2F1, UBE2I, CDKN2A, NOS3, VDR, NR1H2, CAV1, ESR1, TBX3, ESR2, RUNX1, AR, SP1, NME8, PIN1, PPARG, PPARA, CLOCK, TP53, TP73 |
| GO:0010604~positive regulation of macromolecule metabolic process | 22 | 1.93E-10 | CAMK2D, UBE2I, CCNT1, CDKN2A, VDR, NR1H2, CAV1, ESR1, TBX3, ESR2, RUNX1, AR, RXRA, SP1, FAS, PIN1, PPARG, PPARA, CLOCK, TP53, TP63, TP73 |
| GO:0009892~negative regulation of metabolic process | 21 | 2.08E-10 | UBE2I, POU2F1, CCNT1, CDKN2A, VDR, NR1H2, CAV1, ESR1, TBX3, ESR2, RUNX1, SNX3, AR, RXRA, PIN1, PPARG, PPARA, CLOCK, TP53, TP63, TP73 |
| GO:0031327~negative regulation of cellular biosynthetic process | 17 | 5.41E-10 | UBE2I, POU2F1, CDKN2A, VDR, NR1H2, CAV1, ESR1, TBX3, ESR2, RUNX1, RXRA, PPARG, PPARA, CLOCK, TP53, TP63, TP73 |
| GO:0048518~positive regulation of biological process | 27 | 5.52E-10 | CAMK2D, CCNT1, SNX3, RXRA, UBD, TP63, ABCA1, UBE2I, CDKN2A, NOS3, VDR, NR1H2, CAV1, ESR1, TBX3, ESR2, RUNX1, BCR, AR, SP1, FAS, PIN1, PPARG, PPARA, CLOCK, TP53, TP73 |
| GO:0048545~response to steroid hormone | 11 | 6.45E-10 | AR, RXRA, VDR, NR1H2, CAV1, FAS, PPARG, PPARA, ESR1, CLOCK, ESR2 |
| GO:0043401~steroid hormone mediated signaling pathway | 9 | 7.14E-10 | AR, RXRA, VDR, NR1H2, PPARG, PPARA, ESR1, CLOCK, ESR2 |
| GO:0010941~regulation of cell death | 17 | 7.20E-10 | CAMK2D, CDKN2A, NOS3, VDR, CAV1, ESR1, TBX3, AR, UBD, NME8, FAS, PIN1, PPARG, PPARA, TP53, TP63, TP73 |
| GO:0033993~response to lipid | 14 | 8.11E-10 | ABCA1, NOS3, VDR, NR1H2, CAV1, ESR1, ESR2, BCR, AR, RXRA, FAS, PPARG, PPARA, CLOCK |
| GO:0009966~regulation of signal transduction | 21 | 8.78E-10 | ABCA1, CAMK2D, UBE2I, CDKN2A, NOS3, NR1H2, CAV1, ESR1, ESR2, BCR, SNX3, AR, UBD, NME8, FAS, PIN1, PPARG, CLOCK, TP53, TP63, TP73 |
| GO:0034654~nucleobase-containing compound biosynthetic process | 25 | 8.90E-10 | CAMK2D, CCNT1, RXRA, TP63, ABCA1, POU2F1, UBE2I, CDKN2A, NOS3, VDR, NR1H2, CAV1, ESR1, TBX3, ESR2, RUNX1, AR, SP1, NME8, PIN1, PPARG, PPARA, CLOCK, TP53, TP73 |
| GO:0018130~heterocycle biosynthetic process | 25 | 1.17E-09 | CAMK2D, CCNT1, RXRA, TP63, ABCA1, POU2F1, UBE2I, CDKN2A, NOS3, VDR, NR1H2, CAV1, ESR1, TBX3, ESR2, RUNX1, AR, SP1, NME8, PIN1, PPARG, PPARA, CLOCK, TP53, TP73 |
| GO:0065008~regulation of biological quality | 23 | 1.30E-09 | ABCA1, CAMK2D, CCNT1, CDKN2A, NOS3, VDR, NR1H2, CAV1, ESR1, TBX3, BCR, AR, RXRA, SP1, NME8, FAS, PIN1, PPARG, PPARA, CLOCK, TP53, TP63, TP73 |
| GO:0044093~positive regulation of molecular function | 18 | 1.30E-09 | CAMK2D, UBE2I, CCNT1, CDKN2A, NOS3, VDR, NR1H2, CAV1, ESR1, GRIN2B, ESR2, BCR, AR, FAS, PIN1, PPARG, CLOCK, TP73 |
| GO:0065009~regulation of molecular function | 21 | 2.11E-09 | CAMK2D, UBE2I, CCNT1, CDKN2A, NOS3, VDR, NR1H2, CAV1, ESR1, GRIN2B, ESR2, BCR, AR, FAS, PIN1, PPARG, PPARA, CLOCK, TP53, TP63, TP73 |
| GO:0000122~negative regulation of transcription from RNA polymerase II promoter | 13 | 2.31E-09 | UBE2I, VDR, NR1H2, CAV1, ESR1, TBX3, ESR2, RXRA, PPARG, PPARA, TP53, TP63, TP73 |
| GO:0019219~regulation of nucleobase-containing compound metabolic process | 24 | 2.56E-09 | ABCA1, CAMK2D, UBE2I, POU2F1, CCNT1, CDKN2A, NOS3, VDR, NR1H2, CAV1, ESR1, TBX3, ESR2, RUNX1, AR, RXRA, SP1, PIN1, PPARG, PPARA, CLOCK, TP53, TP63, TP73 |
| GO:0042981~regulation of apoptotic process | 16 | 2.83E-09 | CAMK2D, CDKN2A, NOS3, VDR, CAV1, ESR1, TBX3, AR, UBD, NME8, FAS, PIN1, PPARG, TP53, TP63, TP73 |
| GO:0043067~regulation of programmed cell death | 16 | 3.22E-09 | CAMK2D, CDKN2A, NOS3, VDR, CAV1, ESR1, TBX3, AR, UBD, NME8, FAS, PIN1, PPARG, TP53, TP63, TP73 |
| GO:0009755~hormone-mediated signaling pathway | 9 | 3.62E-09 | AR, RXRA, VDR, NR1H2, PPARG, PPARA, ESR1, CLOCK, ESR2 |
| GO:0051239~regulation of multicellular organismal process | 20 | 5.43E-09 | ABCA1, CAMK2D, CDKN2A, NOS3, VDR, NR1H2, CAV1, ESR1, TBX3, RUNX1, BCR, SNX3, AR, RXRA, FAS, PIN1, PPARG, CLOCK, TP63, TP73 |
| GO:0010646~regulation of cell communication | 21 | 5.55E-09 | ABCA1, CAMK2D, UBE2I, CDKN2A, NOS3, NR1H2, CAV1, ESR1, ESR2, BCR, SNX3, AR, UBD, NME8, FAS, PIN1, PPARG, CLOCK, TP53, TP63, TP73 |
| GO:0031324~negative regulation of cellular metabolic process | 19 | 6.17E-09 | UBE2I, POU2F1, CCNT1, CDKN2A, VDR, NR1H2, CAV1, ESR1, TBX3, ESR2, RUNX1, RXRA, PIN1, PPARG, PPARA, CLOCK, TP53, TP63, TP73 |
| GO:0031326~regulation of cellular biosynthetic process | 24 | 6.28E-09 | ABCA1, CAMK2D, UBE2I, POU2F1, CCNT1, CDKN2A, NOS3, VDR, NR1H2, CAV1, ESR1, TBX3, ESR2, RUNX1, AR, RXRA, SP1, PIN1, PPARG, PPARA, CLOCK, TP53, TP63, TP73 |
| GO:0071383~cellular response to steroid hormone stimulus | 9 | 7.14E-09 | AR, RXRA, VDR, NR1H2, PPARG, PPARA, ESR1, CLOCK, ESR2 |
| GO:0023051~regulation of signaling | 21 | 7.45E-09 | ABCA1, CAMK2D, UBE2I, CDKN2A, NOS3, NR1H2, CAV1, ESR1, ESR2, BCR, SNX3, AR, UBD, NME8, FAS, PIN1, PPARG, CLOCK, TP53, TP63, TP73 |
| GO:0009889~regulation of biosynthetic process | 24 | 8.19E-09 | ABCA1, CAMK2D, UBE2I, POU2F1, CCNT1, CDKN2A, NOS3, VDR, NR1H2, CAV1, ESR1, TBX3, ESR2, RUNX1, AR, RXRA, SP1, PIN1, PPARG, PPARA, CLOCK, TP53, TP63, TP73 |
| GO:0071396~cellular response to lipid | 11 | 1.04E-08 | ABCA1, AR, RXRA, VDR, NOS3, NR1H2, PPARG, PPARA, ESR1, CLOCK, ESR2 |
| GO:0045595~regulation of cell differentiation | 16 | 1.09E-08 | ABCA1, CDKN2A, VDR, NR1H2, CAV1, TBX3, RUNX1, SNX3, AR, FAS, PIN1, PPARG, PPARA, CLOCK, TP63, TP73 |
| GO:0051171~regulation of nitrogen compound metabolic process | 24 | 1.11E-08 | ABCA1, CAMK2D, UBE2I, POU2F1, CCNT1, CDKN2A, NOS3, VDR, NR1H2, CAV1, ESR1, TBX3, ESR2, RUNX1, AR, RXRA, SP1, PIN1, PPARG, PPARA, CLOCK, TP53, TP63, TP73 |
| GO:0071407~cellular response to organic cyclic compound | 11 | 1.28E-08 | ABCA1, AR, RXRA, VDR, NR1H2, CAV1, PPARG, PPARA, ESR1, CLOCK, ESR2 |
| GO:0030522~intracellular receptor signaling pathway | 9 | 1.33E-08 | AR, RXRA, VDR, NR1H2, PPARG, PPARA, ESR1, CLOCK, ESR2 |
| GO:0044271~cellular nitrogen compound biosynthetic process | 25 | 1.44E-08 | CAMK2D, CCNT1, RXRA, TP63, ABCA1, POU2F1, UBE2I, CDKN2A, NOS3, VDR, NR1H2, CAV1, ESR1, TBX3, ESR2, RUNX1, AR, SP1, NME8, PIN1, PPARG, PPARA, CLOCK, TP53, TP73 |
| GO:0010033~response to organic substance | 20 | 1.50E-08 | ABCA1, CAMK2D, NOS3, VDR, NR1H2, CAV1, ESR1, GRIN2B, ESR2, BCR, AR, RXRA, SP1, UBD, FAS, PIN1, PPARG, PPARA, CLOCK, TP73 |
| GO:0006355~regulation of transcription, DNA-templated | 22 | 1.62E-08 | CAMK2D, UBE2I, POU2F1, CCNT1, CDKN2A, VDR, NR1H2, CAV1, ESR1, TBX3, ESR2, RUNX1, AR, RXRA, SP1, PIN1, PPARG, PPARA, CLOCK, TP53, TP63, TP73 |
| GO:0006351~transcription, DNA-templated | 22 | 1.64E-08 | CAMK2D, UBE2I, POU2F1, CCNT1, CDKN2A, VDR, NR1H2, CAV1, ESR1, TBX3, ESR2, RUNX1, AR, RXRA, SP1, PIN1, PPARG, PPARA, CLOCK, TP53, TP63, TP73 |
| GO:1903506~regulation of nucleic acid-templated transcription | 22 | 1.81E-08 | CAMK2D, UBE2I, POU2F1, CCNT1, CDKN2A, VDR, NR1H2, CAV1, ESR1, TBX3, ESR2, RUNX1, AR, RXRA, SP1, PIN1, PPARG, PPARA, CLOCK, TP53, TP63, TP73 |
| GO:0006367~transcription initiation from RNA polymerase II promoter | 8 | 1.89E-08 | AR, RXRA, VDR, NR1H2, PPARG, PPARA, ESR1, ESR2 |
| GO:0048583~regulation of response to stimulus | 22 | 1.92E-08 | ABCA1, CAMK2D, UBE2I, CDKN2A, NOS3, NR1H2, CAV1, ESR1, ESR2, BCR, SNX3, AR, UBD, NME8, FAS, PIN1, PPARG, PPARA, CLOCK, TP53, TP63, TP73 |
| GO:2001141~regulation of RNA biosynthetic process | 22 | 2.00E-08 | CAMK2D, UBE2I, POU2F1, CCNT1, CDKN2A, VDR, NR1H2, CAV1, ESR1, TBX3, ESR2, RUNX1, AR, RXRA, SP1, PIN1, PPARG, PPARA, CLOCK, TP53, TP63, TP73 |
| GO:0030154~cell differentiation | 22 | 2.09E-08 | ABCA1, CAMK2D, CDKN2A, VDR, NR1H2, CAV1, ESR1, TBX3, RUNX1, SNX3, AR, RXRA, UBD, NME8, FAS, PIN1, PPARG, PPARA, CLOCK, TP53, TP63, TP73 |
| GO:0048585~negative regulation of response to stimulus | 15 | 2.24E-08 | NOS3, NR1H2, CAV1, ESR1, ESR2, BCR, AR, NME8, FAS, PIN1, PPARG, PPARA, CLOCK, TP63, TP73 |
| GO:0044700~single organism signaling | 27 | 2.66E-08 | CAMK2D, SNX3, RXRA, UBD, TP63, ABCA1, UBE2I, CDKN2A, NOS3, VDR, NR1H2, CAV1, GRIN2B, ESR1, TBX3, ESR2, RUNX1, BCR, AR, NME8, FAS, PIN1, PPARG, PPARA, CLOCK, TP53, TP73 |
| GO:0043065~positive regulation of apoptotic process | 11 | 3.16E-08 | CAMK2D, CDKN2A, VDR, CAV1, UBD, FAS, PIN1, PPARG, TP53, TP63, TP73 |
| GO:0008219~cell death | 17 | 3.21E-08 | CAMK2D, CDKN2A, NOS3, VDR, CAV1, ESR1, TBX3, AR, UBD, NME8, FAS, PIN1, PPARG, PPARA, TP53, TP63, TP73 |
| GO:0023052~signaling | 27 | 3.26E-08 | CAMK2D, SNX3, RXRA, UBD, TP63, ABCA1, UBE2I, CDKN2A, NOS3, VDR, NR1H2, CAV1, GRIN2B, ESR1, TBX3, ESR2, RUNX1, BCR, AR, NME8, FAS, PIN1, PPARG, PPARA, CLOCK, TP53, TP73 |
| GO:0043068~positive regulation of programmed cell death | 11 | 3.43E-08 | CAMK2D, CDKN2A, VDR, CAV1, UBD, FAS, PIN1, PPARG, TP53, TP63, TP73 |
| GO:0007154~cell communication | 27 | 3.45E-08 | CAMK2D, SNX3, RXRA, UBD, TP63, ABCA1, UBE2I, CDKN2A, NOS3, VDR, NR1H2, CAV1, GRIN2B, ESR1, TBX3, ESR2, RUNX1, BCR, AR, NME8, FAS, PIN1, PPARG, PPARA, CLOCK, TP53, TP73 |
| GO:0051716~cellular response to stimulus | 28 | 3.58E-08 | CAMK2D, SNX3, RXRA, UBD, TP63, ABCA1, UBE2I, CDKN2A, NOS3, VDR, NR1H2, CAV1, GRIN2B, ESR1, TBX3, ESR2, RUNX1, BCR, AR, SP1, NME8, FAS, PIN1, PPARG, PPARA, CLOCK, TP53, TP73 |
| GO:0051252~regulation of RNA metabolic process | 22 | 3.63E-08 | CAMK2D, UBE2I, POU2F1, CCNT1, CDKN2A, VDR, NR1H2, CAV1, ESR1, TBX3, ESR2, RUNX1, AR, RXRA, SP1, PIN1, PPARG, PPARA, CLOCK, TP53, TP63, TP73 |
| GO:0097659~nucleic acid-templated transcription | 22 | 3.79E-08 | CAMK2D, UBE2I, POU2F1, CCNT1, CDKN2A, VDR, NR1H2, CAV1, ESR1, TBX3, ESR2, RUNX1, AR, RXRA, SP1, PIN1, PPARG, PPARA, CLOCK, TP53, TP63, TP73 |
| GO:2000026~regulation of multicellular organismal development | 16 | 4.74E-08 | CDKN2A, NOS3, VDR, CAV1, ESR1, TBX3, RUNX1, SNX3, AR, RXRA, FAS, PIN1, PPARG, CLOCK, TP63, TP73 |
| GO:0007165~signal transduction | 26 | 4.82E-08 | CAMK2D, SNX3, RXRA, UBD, TP63, ABCA1, UBE2I, CDKN2A, NOS3, VDR, NR1H2, CAV1, GRIN2B, ESR1, ESR2, RUNX1, BCR, AR, NME8, FAS, PIN1, PPARG, PPARA, CLOCK, TP53, TP73 |
| GO:0051704~multi-organism process | 18 | 5.25E-08 | ABCA1, UBE2I, CCNT1, NOS3, VDR, CAV1, ESR1, BCR, SNX3, AR, RXRA, SP1, NME8, FAS, CLOCK, TP53, TP63, TP73 |
| GO:0080090~regulation of primary metabolic process | 26 | 5.27E-08 | CAMK2D, CCNT1, SNX3, RXRA, TP63, ABCA1, POU2F1, UBE2I, CDKN2A, NOS3, VDR, NR1H2, CAV1, ESR1, TBX3, ESR2, RUNX1, AR, SP1, FAS, PIN1, PPARG, PPARA, CLOCK, TP53, TP73 |
| GO:0010942~positive regulation of cell death | 11 | 5.53E-08 | CAMK2D, CDKN2A, VDR, CAV1, UBD, FAS, PIN1, PPARG, TP53, TP63, TP73 |
| GO:0050790~regulation of catalytic activity | 18 | 5.82E-08 | CAMK2D, UBE2I, CCNT1, CDKN2A, NOS3, VDR, NR1H2, CAV1, ESR1, GRIN2B, BCR, AR, FAS, PIN1, PPARG, TP53, TP63, TP73 |
| GO:0032870~cellular response to hormone stimulus | 11 | 6.35E-08 | AR, RXRA, SP1, VDR, NR1H2, CAV1, PPARG, PPARA, ESR1, CLOCK, ESR2 |
| GO:0032774~RNA biosynthetic process | 22 | 7.25E-08 | CAMK2D, UBE2I, POU2F1, CCNT1, CDKN2A, VDR, NR1H2, CAV1, ESR1, TBX3, ESR2, RUNX1, AR, RXRA, SP1, PIN1, PPARG, PPARA, CLOCK, TP53, TP63, TP73 |
| GO:0006725~cellular aromatic compound metabolic process | 26 | 9.99E-08 | PNMT, CAMK2D, CCNT1, RXRA, TP63, ABCA1, POU2F1, UBE2I, CDKN2A, NOS3, VDR, NR1H2, CAV1, ESR1, TBX3, ESR2, RUNX1, AR, SP1, NME8, PIN1, PPARG, PPARA, CLOCK, TP53, TP73 |
| GO:0010887~negative regulation of cholesterol storage | 4 | 1.03E-07 | ABCA1, NR1H2, PPARG, PPARA |
| GO:0050793~regulation of developmental process | 17 | 1.06E-07 | CDKN2A, NOS3, VDR, CAV1, ESR1, TBX3, RUNX1, BCR, SNX3, AR, RXRA, FAS, PIN1, PPARG, CLOCK, TP63, TP73 |
| GO:0009719~response to endogenous stimulus | 15 | 1.06E-07 | ABCA1, VDR, NR1H2, CAV1, ESR1, ESR2, AR, RXRA, SP1, FAS, PIN1, PPARG, PPARA, CLOCK, TP73 |
| GO:2000112~regulation of cellular macromolecule biosynthetic process | 22 | 1.08E-07 | CAMK2D, UBE2I, POU2F1, CCNT1, CDKN2A, VDR, NR1H2, CAV1, ESR1, TBX3, ESR2, RUNX1, AR, RXRA, SP1, PIN1, PPARG, PPARA, CLOCK, TP53, TP63, TP73 |
| GO:0048869~cellular developmental process | 22 | 1.09E-07 | ABCA1, CAMK2D, CDKN2A, VDR, NR1H2, CAV1, ESR1, TBX3, RUNX1, SNX3, AR, RXRA, UBD, NME8, FAS, PIN1, PPARG, PPARA, CLOCK, TP53, TP63, TP73 |
| GO:0043085~positive regulation of catalytic activity | 15 | 1.16E-07 | CAMK2D, UBE2I, CCNT1, CDKN2A, NOS3, VDR, NR1H2, CAV1, ESR1, GRIN2B, BCR, FAS, PIN1, PPARG, TP73 |
| GO:0009725~response to hormone | 12 | 1.24E-07 | AR, RXRA, SP1, VDR, NR1H2, CAV1, FAS, PPARG, PPARA, ESR1, CLOCK, ESR2 |
| GO:0012501~programmed cell death | 16 | 1.27E-07 | CAMK2D, CDKN2A, NOS3, VDR, CAV1, ESR1, TBX3, AR, UBD, NME8, FAS, PIN1, PPARG, TP53, TP63, TP73 |
| GO:0006352~DNA-templated transcription, initiation | 8 | 1.32E-07 | AR, RXRA, VDR, NR1H2, PPARG, PPARA, ESR1, ESR2 |
| GO:0044249~cellular biosynthetic process | 26 | 1.76E-07 | PNMT, CAMK2D, CCNT1, RXRA, TP63, ABCA1, POU2F1, UBE2I, CDKN2A, NOS3, VDR, NR1H2, CAV1, ESR1, TBX3, ESR2, RUNX1, AR, SP1, NME8, PIN1, PPARG, PPARA, CLOCK, TP53, TP73 |
| GO:0010556~regulation of macromolecule biosynthetic process | 22 | 1.84E-07 | CAMK2D, UBE2I, POU2F1, CCNT1, CDKN2A, VDR, NR1H2, CAV1, ESR1, TBX3, ESR2, RUNX1, AR, RXRA, SP1, PIN1, PPARG, PPARA, CLOCK, TP53, TP63, TP73 |
| GO:0042221~response to chemical | 22 | 1.92E-07 | ABCA1, CAMK2D, NOS3, VDR, NR1H2, CAV1, ESR1, GRIN2B, ESR2, BCR, AR, RXRA, SP1, UBD, NME8, FAS, PIN1, PPARG, PPARA, CLOCK, TP53, TP73 |
| GO:1901360~organic cyclic compound metabolic process | 26 | 1.97E-07 | PNMT, CAMK2D, CCNT1, RXRA, TP63, ABCA1, POU2F1, UBE2I, CDKN2A, NOS3, VDR, NR1H2, CAV1, ESR1, TBX3, ESR2, RUNX1, AR, SP1, NME8, PIN1, PPARG, PPARA, CLOCK, TP53, TP73 |
| GO:0019222~regulation of metabolic process | 26 | 2.14E-07 | CAMK2D, CCNT1, SNX3, RXRA, TP63, ABCA1, POU2F1, UBE2I, CDKN2A, NOS3, VDR, NR1H2, CAV1, ESR1, TBX3, ESR2, RUNX1, AR, SP1, FAS, PIN1, PPARG, PPARA, CLOCK, TP53, TP73 |
| GO:0014070~response to organic cyclic compound | 12 | 2.25E-07 | ABCA1, AR, RXRA, VDR, NR1H2, CAV1, FAS, PPARG, PPARA, ESR1, CLOCK, ESR2 |
| GO:1901576~organic substance biosynthetic process | 26 | 2.62E-07 | PNMT, CAMK2D, CCNT1, RXRA, TP63, ABCA1, POU2F1, UBE2I, CDKN2A, NOS3, VDR, NR1H2, CAV1, ESR1, TBX3, ESR2, RUNX1, AR, SP1, NME8, PIN1, PPARG, PPARA, CLOCK, TP53, TP73 |
| GO:0032270~positive regulation of cellular protein metabolic process | 14 | 3.26E-07 | CAMK2D, UBE2I, CCNT1, CDKN2A, NR1H2, CAV1, ESR1, AR, RXRA, FAS, PIN1, PPARG, TP53, TP73 |
| GO:0070887~cellular response to chemical stimulus | 18 | 3.31E-07 | ABCA1, CAMK2D, NOS3, VDR, NR1H2, CAV1, ESR1, ESR2, AR, RXRA, SP1, NME8, FAS, PIN1, PPARG, PPARA, CLOCK, TP53 |
| GO:0009058~biosynthetic process | 26 | 3.64E-07 | PNMT, CAMK2D, CCNT1, RXRA, TP63, ABCA1, POU2F1, UBE2I, CDKN2A, NOS3, VDR, NR1H2, CAV1, ESR1, TBX3, ESR2, RUNX1, AR, SP1, NME8, PIN1, PPARG, PPARA, CLOCK, TP53, TP73 |
| GO:1902531~regulation of intracellular signal transduction | 15 | 3.79E-07 | ABCA1, CAMK2D, UBE2I, CDKN2A, CAV1, ESR1, BCR, AR, UBD, NME8, FAS, PIN1, TP53, TP63, TP73 |
| GO:0006139~nucleobase-containing compound metabolic process | 25 | 4.23E-07 | CAMK2D, CCNT1, RXRA, TP63, ABCA1, POU2F1, UBE2I, CDKN2A, NOS3, VDR, NR1H2, CAV1, ESR1, TBX3, ESR2, RUNX1, AR, SP1, NME8, PIN1, PPARG, PPARA, CLOCK, TP53, TP73 |
| GO:0006915~apoptotic process | 15 | 4.80E-07 | CAMK2D, CDKN2A, NOS3, VDR, CAV1, TBX3, AR, UBD, NME8, FAS, PIN1, PPARG, TP53, TP63, TP73 |
| GO:0031323~regulation of cellular metabolic process | 25 | 5.07E-07 | CAMK2D, CCNT1, RXRA, TP63, ABCA1, POU2F1, UBE2I, CDKN2A, NOS3, VDR, NR1H2, CAV1, ESR1, TBX3, ESR2, RUNX1, AR, SP1, FAS, PIN1, PPARG, PPARA, CLOCK, TP53, TP73 |
| GO:0010468~regulation of gene expression | 22 | 5.13E-07 | CAMK2D, UBE2I, POU2F1, CCNT1, CDKN2A, VDR, NR1H2, CAV1, ESR1, TBX3, ESR2, RUNX1, AR, RXRA, SP1, PIN1, PPARG, PPARA, CLOCK, TP53, TP63, TP73 |
| GO:0051247~positive regulation of protein metabolic process | 14 | 6.54E-07 | CAMK2D, UBE2I, CCNT1, CDKN2A, NR1H2, CAV1, ESR1, AR, RXRA, FAS, PIN1, PPARG, TP53, TP73 |
| GO:0046483~heterocycle metabolic process | 25 | 6.63E-07 | CAMK2D, CCNT1, RXRA, TP63, ABCA1, POU2F1, UBE2I, CDKN2A, NOS3, VDR, NR1H2, CAV1, ESR1, TBX3, ESR2, RUNX1, AR, SP1, NME8, PIN1, PPARG, PPARA, CLOCK, TP53, TP73 |
| GO:0044707~single-multicellular organism process | 25 | 7.22E-07 | CAMK2D, SNX3, RXRA, UBD, TP63, ABCA1, CDKN2A, NOS3, VDR, NR1H2, CAV1, GRIN2B, ESR1, TBX3, RUNX1, BCR, AR, NME8, FAS, PIN1, PPARG, PPARA, CLOCK, TP53, TP73 |
| GO:0080134~regulation of response to stress | 13 | 9.05E-07 | CAMK2D, CDKN2A, NOS3, NR1H2, CAV1, ESR1, BCR, NME8, PPARG, PPARA, CLOCK, TP63, TP73 |
| GO:0048511~rhythmic process | 8 | 1.01E-06 | SP1, NOS3, FAS, PPARG, PPARA, ESR1, TP53, CLOCK |
| GO:0060443~mammary gland morphogenesis | 5 | 1.07E-06 | AR, VDR, CAV1, ESR1, TBX3 |
| GO:0034645~cellular macromolecule biosynthetic process | 23 | 1.10E-06 | ABCA1, CAMK2D, UBE2I, POU2F1, CCNT1, CDKN2A, VDR, NR1H2, CAV1, ESR1, TBX3, ESR2, RUNX1, AR, RXRA, SP1, PIN1, PPARG, PPARA, CLOCK, TP53, TP63, TP73 |
| GO:0045596~negative regulation of cell differentiation | 10 | 1.12E-06 | ABCA1, CDKN2A, NR1H2, CAV1, PPARG, PPARA, TP63, TBX3, TP73, RUNX1 |
| GO:0071310~cellular response to organic substance | 16 | 1.13E-06 | ABCA1, CAMK2D, NOS3, VDR, NR1H2, CAV1, ESR1, ESR2, AR, RXRA, SP1, FAS, PIN1, PPARG, PPARA, CLOCK |
| GO:0071704~organic substance metabolic process | 31 | 1.35E-06 | PNMT, CAMK2D, CCNT1, SNX3, RXRA, UBD, TP63, ABCA1, POU2F1, UBE2I, CDKN2A, NOS3, VDR, NR1H2, CAV1, GRIN2B, ESR1, TBX3, ESR2, RUNX1, BCR, AR, SP1, NME8, FAS, PIN1, PPARG, PPARA, CLOCK, TP53, TP73 |
| GO:0051336~regulation of hydrolase activity | 13 | 1.38E-06 | CAMK2D, CDKN2A, NOS3, NR1H2, CAV1, ESR1, GRIN2B, BCR, FAS, PIN1, PPARG, TP53, TP63 |
| GO:0009968~negative regulation of signal transduction | 12 | 1.41E-06 | AR, NOS3, NR1H2, NME8, CAV1, FAS, PIN1, PPARG, ESR1, CLOCK, ESR2, TP73 |
| GO:0010745~negative regulation of macrophage-derived foam cell differentiation | 4 | 1.45E-06 | ABCA1, NR1H2, PPARG, PPARA |
| GO:0010885~regulation of cholesterol storage | 4 | 1.45E-06 | ABCA1, NR1H2, PPARG, PPARA |
| GO:0022612~gland morphogenesis | 6 | 1.72E-06 | AR, VDR, CAV1, ESR1, TP63, TBX3 |
| GO:0016070~RNA metabolic process | 22 | 1.72E-06 | CAMK2D, UBE2I, POU2F1, CCNT1, CDKN2A, VDR, NR1H2, CAV1, ESR1, TBX3, ESR2, RUNX1, AR, RXRA, SP1, PIN1, PPARG, PPARA, CLOCK, TP53, TP63, TP73 |
| GO:0009059~macromolecule biosynthetic process | 23 | 2.01E-06 | ABCA1, CAMK2D, UBE2I, POU2F1, CCNT1, CDKN2A, VDR, NR1H2, CAV1, ESR1, TBX3, ESR2, RUNX1, AR, RXRA, SP1, PIN1, PPARG, PPARA, CLOCK, TP53, TP63, TP73 |
| GO:0060548~negative regulation of cell death | 11 | 2.03E-06 | AR, NOS3, NME8, CAV1, FAS, PIN1, PPARA, TP53, TP63, TBX3, TP73 |
| GO:0010878~cholesterol storage | 4 | 2.31E-06 | ABCA1, NR1H2, PPARG, PPARA |
| GO:0035556~intracellular signal transduction | 17 | 2.31E-06 | ABCA1, CAMK2D, UBE2I, CDKN2A, NOS3, CAV1, ESR1, GRIN2B, BCR, AR, UBD, NME8, FAS, PIN1, TP53, TP63, TP73 |
| GO:0006807~nitrogen compound metabolic process | 26 | 2.92E-06 | PNMT, CAMK2D, CCNT1, RXRA, TP63, ABCA1, POU2F1, UBE2I, CDKN2A, NOS3, VDR, NR1H2, CAV1, ESR1, TBX3, ESR2, RUNX1, AR, SP1, NME8, PIN1, PPARG, PPARA, CLOCK, TP53, TP73 |
| GO:0060255~regulation of macromolecule metabolic process | 24 | 3.06E-06 | CAMK2D, UBE2I, POU2F1, CCNT1, CDKN2A, VDR, NR1H2, CAV1, ESR1, TBX3, ESR2, RUNX1, SNX3, AR, RXRA, SP1, FAS, PIN1, PPARG, PPARA, CLOCK, TP53, TP63, TP73 |
| GO:0010648~negative regulation of cell communication | 12 | 3.15E-06 | AR, NOS3, NR1H2, NME8, CAV1, FAS, PIN1, PPARG, ESR1, CLOCK, ESR2, TP73 |
| GO:0010467~gene expression | 23 | 3.20E-06 | CAMK2D, UBE2I, POU2F1, CCNT1, CDKN2A, VDR, NR1H2, CAV1, ESR1, TBX3, ESR2, RUNX1, AR, RXRA, SP1, FAS, PIN1, PPARG, PPARA, CLOCK, TP53, TP63, TP73 |
| GO:0071495~cellular response to endogenous stimulus | 12 | 3.23E-06 | AR, RXRA, SP1, VDR, NR1H2, CAV1, PIN1, PPARG, PPARA, ESR1, CLOCK, ESR2 |
| GO:0023057~negative regulation of signaling | 12 | 3.25E-06 | AR, NOS3, NR1H2, NME8, CAV1, FAS, PIN1, PPARG, ESR1, CLOCK, ESR2, TP73 |
| GO:0050896~response to stimulus | 28 | 3.41E-06 | CAMK2D, SNX3, RXRA, UBD, TP63, ABCA1, UBE2I, CDKN2A, NOS3, VDR, NR1H2, CAV1, GRIN2B, ESR1, TBX3, ESR2, RUNX1, BCR, AR, SP1, NME8, FAS, PIN1, PPARG, PPARA, CLOCK, TP53, TP73 |
| GO:0010888~negative regulation of lipid storage | 4 | 3.44E-06 | ABCA1, NR1H2, PPARG, PPARA |
| GO:1901700~response to oxygen-containing compound | 13 | 3.65E-06 | ABCA1, NOS3, VDR, CAV1, ESR1, GRIN2B, BCR, RXRA, SP1, NME8, FAS, PPARG, PPARA |
| GO:0009888~tissue development | 14 | 3.70E-06 | CAMK2D, VDR, CAV1, ESR1, TBX3, RUNX1, AR, RXRA, PIN1, PPARG, PPARA, CLOCK, TP63, TP73 |
| GO:0008152~metabolic process | 31 | 3.73E-06 | PNMT, CAMK2D, CCNT1, SNX3, RXRA, UBD, TP63, ABCA1, POU2F1, UBE2I, CDKN2A, NOS3, VDR, NR1H2, CAV1, GRIN2B, ESR1, TBX3, ESR2, RUNX1, BCR, AR, SP1, NME8, FAS, PIN1, PPARG, PPARA, CLOCK, TP53, TP73 |
| GO:2001233~regulation of apoptotic signaling pathway | 8 | 3.74E-06 | AR, NOS3, NME8, CAV1, FAS, TP53, TP63, TP73 |
| GO:0032501~multicellular organismal process | 26 | 3.74E-06 | CAMK2D, SNX3, RXRA, UBD, TP63, ABCA1, CDKN2A, NOS3, VDR, NR1H2, CAV1, GRIN2B, ESR1, TBX3, RUNX1, BCR, AR, SP1, NME8, FAS, PIN1, PPARG, PPARA, CLOCK, TP53, TP73 |
| GO:0048513~animal organ development | 18 | 3.76E-06 | CAMK2D, CDKN2A, NOS3, VDR, CAV1, ESR1, TBX3, RUNX1, BCR, AR, RXRA, UBD, FAS, PIN1, PPARG, PPARA, TP63, TP73 |
| GO:0019915~lipid storage | 5 | 3.76E-06 | ABCA1, NR1H2, CAV1, PPARG, PPARA |
| GO:0007275~multicellular organism development | 22 | 4.04E-06 | CAMK2D, CDKN2A, NOS3, VDR, CAV1, ESR1, TBX3, RUNX1, BCR, SNX3, AR, RXRA, UBD, NME8, FAS, PIN1, PPARG, PPARA, CLOCK, TP53, TP63, TP73 |
| GO:0035265~organ growth | 6 | 4.21E-06 | AR, CAMK2D, RXRA, PIN1, ESR1, TP73 |
| GO:0030855~epithelial cell differentiation | 9 | 4.63E-06 | AR, RXRA, VDR, CAV1, PPARG, ESR1, CLOCK, TP63, TBX3 |
| GO:0034641~cellular nitrogen compound metabolic process | 25 | 4.71E-06 | CAMK2D, CCNT1, RXRA, TP63, ABCA1, POU2F1, UBE2I, CDKN2A, NOS3, VDR, NR1H2, CAV1, ESR1, TBX3, ESR2, RUNX1, AR, SP1, NME8, PIN1, PPARG, PPARA, CLOCK, TP53, TP73 |
| GO:0051049~regulation of transport | 14 | 4.76E-06 | ABCA1, CAMK2D, CDKN2A, NOS3, NR1H2, CAV1, BCR, SNX3, PPARG, PPARA, CLOCK, TP53, TP63, TP73 |
| GO:0032879~regulation of localization | 16 | 5.48E-06 | ABCA1, CAMK2D, CDKN2A, NOS3, NR1H2, CAV1, BCR, SNX3, AR, PIN1, PPARG, PPARA, CLOCK, TP53, TP63, TP73 |
| GO:0044237~cellular metabolic process | 30 | 6.46E-06 | PNMT, CAMK2D, CCNT1, RXRA, UBD, TP63, ABCA1, POU2F1, UBE2I, CDKN2A, NOS3, VDR, NR1H2, CAV1, GRIN2B, ESR1, TBX3, ESR2, RUNX1, BCR, AR, SP1, NME8, FAS, PIN1, PPARG, PPARA, CLOCK, TP53, TP73 |
| GO:0042127~regulation of cell proliferation | 13 | 6.62E-06 | CAMK2D, CDKN2A, NOS3, VDR, CAV1, ESR1, TBX3, AR, FAS, PPARG, TP53, TP63, TP73 |
| GO:0051246~regulation of protein metabolic process | 16 | 6.68E-06 | CAMK2D, UBE2I, CCNT1, CDKN2A, NR1H2, CAV1, ESR1, SNX3, AR, RXRA, FAS, PIN1, PPARG, TP53, TP63, TP73 |
| GO:0060444~branching involved in mammary gland duct morphogenesis | 4 | 6.70E-06 | AR, VDR, ESR1, TBX3 |
| GO:0048608~reproductive structure development | 8 | 6.99E-06 | AR, RXRA, VDR, NOS3, PPARG, ESR1, TP63, TBX3 |
| GO:0006950~response to stress | 19 | 7.06E-06 | ABCA1, CAMK2D, UBE2I, CDKN2A, NOS3, NR1H2, CAV1, ESR1, TBX3, BCR, UBD, NME8, FAS, PPARG, PPARA, CLOCK, TP53, TP63, TP73 |
| GO:0008283~cell proliferation | 14 | 7.08E-06 | CAMK2D, CDKN2A, NOS3, VDR, CAV1, ESR1, TBX3, RUNX1, AR, RXRA, PPARG, TP53, TP63, TP73 |
| GO:0044238~primary metabolic process | 30 | 7.22E-06 | CAMK2D, CCNT1, SNX3, RXRA, UBD, TP63, ABCA1, POU2F1, UBE2I, CDKN2A, NOS3, VDR, NR1H2, CAV1, GRIN2B, ESR1, TBX3, ESR2, RUNX1, BCR, AR, SP1, NME8, FAS, PIN1, PPARG, PPARA, CLOCK, TP53, TP73 |
| GO:0061458~reproductive system development | 8 | 7.55E-06 | AR, RXRA, VDR, NOS3, PPARG, ESR1, TP63, TBX3 |
| GO:0043066~negative regulation of apoptotic process | 10 | 8.38E-06 | AR, NOS3, NME8, CAV1, FAS, PIN1, TP53, TP63, TBX3, TP73 |
| GO:0043069~negative regulation of programmed cell death | 10 | 9.34E-06 | AR, NOS3, NME8, CAV1, FAS, PIN1, TP53, TP63, TBX3, TP73 |

**Table S9.** Biological processes related to the third group of genes (Cluster 3)

| **Term** | **Count** | **PValue** | **Genes** |
| --- | --- | --- | --- |
| GO:0033619~membrane protein proteolysis | 9 | 1.78E-15 | APH1A, BACE1, PSENEN, BACE2, NCSTN, TGFB1, APH1B, PSEN2, PSEN1 |
| GO:0007220~Notch receptor processing | 6 | 7.92E-12 | APH1A, PSENEN, NCSTN, APH1B, PSEN2, PSEN1 |
| GO:0006509~membrane protein ectodomain proteolysis | 7 | 8.90E-12 | APH1A, BACE1, PSENEN, BACE2, NCSTN, PSEN2, PSEN1 |
| GO:0031293~membrane protein intracellular domain proteolysis | 6 | 1.55E-11 | APH1A, PSENEN, NCSTN, TGFB1, APH1B, PSEN2 |
| GO:0042982~amyloid precursor protein metabolic process | 6 | 3.05E-10 | APH1A, PSENEN, BACE2, NCSTN, PSEN2, PSEN1 |
| GO:0007219~Notch signaling pathway | 8 | 7.09E-10 | APH1A, PSENEN, APP, NCSTN, TGFB1, APH1B, PSEN2, PSEN1 |
| GO:0006915~apoptotic process | 15 | 9.51E-10 | PSENEN, APP, TGFB1, PSEN2, PSEN1, MAPK8IP1, APH1A, NCSTN, APH1B, UBQLN1, APBB2, CD14, APBB1, TARDBP, TLR4 |
| GO:0012501~programmed cell death | 15 | 2.06E-09 | PSENEN, APP, TGFB1, PSEN2, PSEN1, MAPK8IP1, APH1A, NCSTN, APH1B, UBQLN1, APBB2, CD14, APBB1, TARDBP, TLR4 |
| GO:0008219~cell death | 15 | 4.31E-09 | PSENEN, APP, TGFB1, PSEN2, PSEN1, MAPK8IP1, APH1A, NCSTN, APH1B, UBQLN1, APBB2, CD14, APBB1, TARDBP, TLR4 |
| GO:0042987~amyloid precursor protein catabolic process | 5 | 8.64E-09 | APH1A, PSENEN, NCSTN, PSEN2, PSEN1 |
| GO:0050435~beta-amyloid metabolic process | 5 | 1.89E-08 | BACE1, BACE2, NCSTN, PSEN2, PSEN1 |
| GO:0043065~positive regulation of apoptotic process | 9 | 2.37E-07 | APH1A, PSENEN, NCSTN, TGFB1, APH1B, PSEN2, APBB2, APBB1, PSEN1 |
| GO:0043068~positive regulation of programmed cell death | 9 | 2.54E-07 | APH1A, PSENEN, NCSTN, TGFB1, APH1B, PSEN2, APBB2, APBB1, PSEN1 |
| GO:0016485~protein processing | 7 | 3.29E-07 | APH1A, PSENEN, BACE2, NCSTN, APH1B, PSEN2, PSEN1 |
| GO:0010942~positive regulation of cell death | 9 | 3.73E-07 | APH1A, PSENEN, NCSTN, TGFB1, APH1B, PSEN2, APBB2, APBB1, PSEN1 |
| GO:0051604~protein maturation | 7 | 7.40E-07 | APH1A, PSENEN, BACE2, NCSTN, APH1B, PSEN2, PSEN1 |
| GO:0006508~proteolysis | 12 | 1.43E-06 | APH1A, BACE1, PSENEN, BACE2, APP, NCSTN, TGFB1, APH1B, PSEN2, UBQLN1, PSEN1, CTSD |
| GO:0042981~regulation of apoptotic process | 11 | 2.36E-06 | APH1A, PSENEN, NCSTN, TGFB1, APH1B, PSEN2, UBQLN1, APBB2, APBB1, PSEN1, MAPK8IP1 |
| GO:0043067~regulation of programmed cell death | 11 | 2.56E-06 | APH1A, PSENEN, NCSTN, TGFB1, APH1B, PSEN2, UBQLN1, APBB2, APBB1, PSEN1, MAPK8IP1 |
| GO:0048013~ephrin receptor signaling pathway | 5 | 3.76E-06 | APH1A, PSENEN, NCSTN, APH1B, PSEN2 |
| GO:0010941~regulation of cell death | 11 | 4.66E-06 | APH1A, PSENEN, NCSTN, TGFB1, APH1B, PSEN2, UBQLN1, APBB2, APBB1, PSEN1, MAPK8IP1 |
| GO:0007169~transmembrane receptor protein tyrosine kinase signaling pathway | 8 | 8.94E-06 | APH1A, PSENEN, APP, NCSTN, TGFB1, APH1B, PSEN2, PSEN1 |
| GO:0019538~protein metabolic process | 17 | 5.06E-05 | PSENEN, APP, TGFB1, PSEN2, PSEN1, MAPK8IP1, TRAK2, APH1A, BACE1, BACE2, NCSTN, APH1B, UBQLN1, APBB1, TARDBP, CTSD, TLR4 |

**Table S10.** Biological processes related to the sixth group of genes (Cluster 6)

| **Term** | **Count** | **PValue** | **Genes** |
| --- | --- | --- | --- |
| GO:0048519~negative regulation of biological process | 18 | 3.91E-09 | GSK3B, SERPINA1, DAPK1, GSTP1, LRRK2, EIF2AK2, UBE2D1, TRAF2, TNF, SLC6A3, SLC6A4, LRP6, RCAN1, YWHAQ, EIF4EBP1, NLRP3, RAB7A, SNCA |
| GO:0048585~negative regulation of response to stimulus | 12 | 1.89E-08 | GSK3B, DAPK1, GSTP1, LRRK2, NLRP3, EIF2AK2, TRAF2, TNF, SLC6A3, RAB7A, LRP6, SNCA |
| GO:0042981~regulation of apoptotic process | 12 | 2.39E-08 | GSK3B, YWHAQ, DAPK1, GSTP1, LRRK2, NLRP3, EIF2AK2, TRAF2, UNC5C, TNF, LRP6, SNCA |
| GO:0043067~regulation of programmed cell death | 12 | 2.64E-08 | GSK3B, YWHAQ, DAPK1, GSTP1, LRRK2, NLRP3, EIF2AK2, TRAF2, UNC5C, TNF, LRP6, SNCA |
| GO:0048523~negative regulation of cellular process | 17 | 2.96E-08 | GSK3B, SERPINA1, DAPK1, GSTP1, LRRK2, EIF2AK2, UBE2D1, TRAF2, TNF, SLC6A4, LRP6, RCAN1, YWHAQ, EIF4EBP1, NLRP3, RAB7A, SNCA |
| GO:0045862~positive regulation of proteolysis | 8 | 4.91E-08 | GSK3B, DAPK1, LRRK2, NLRP3, UBE2D1, TRAF2, TNF, SNCA |
| GO:0010941~regulation of cell death | 12 | 5.23E-08 | GSK3B, YWHAQ, DAPK1, GSTP1, LRRK2, NLRP3, EIF2AK2, TRAF2, UNC5C, TNF, LRP6, SNCA |
| GO:0043068~positive regulation of programmed cell death | 9 | 5.98E-08 | GSK3B, YWHAQ, DAPK1, LRRK2, NLRP3, TRAF2, UNC5C, TNF, SNCA |
| GO:0051246~regulation of protein metabolic process | 14 | 7.46E-08 | GSK3B, SERPINA1, DAPK1, GSTP1, LRRK2, EIF2AK2, UBE2D1, TRAF2, TNF, LRP6, EIF4EBP1, NLRP3, RAB7A, SNCA |
| GO:0010648~negative regulation of cell communication | 11 | 7.47E-08 | GSK3B, DAPK1, GSTP1, LRRK2, NLRP3, TRAF2, TNF, SLC6A4, RAB7A, LRP6, SNCA |
| GO:0023057~negative regulation of signaling | 11 | 7.71E-08 | GSK3B, DAPK1, GSTP1, LRRK2, NLRP3, TRAF2, TNF, SLC6A4, RAB7A, LRP6, SNCA |
| GO:0010942~positive regulation of cell death | 9 | 8.84E-08 | GSK3B, YWHAQ, DAPK1, LRRK2, NLRP3, TRAF2, UNC5C, TNF, SNCA |
| GO:0071310~cellular response to organic substance | 13 | 2.00E-07 | GSK3B, DAPK1, GSTP1, LRRK2, EIF2AK2, UBE2D1, TRAF2, TNF, SLC6A4, LRP6, EIF4EBP1, NLRP3, SNCA |
| GO:0006915~apoptotic process | 12 | 2.38E-07 | GSK3B, YWHAQ, DAPK1, GSTP1, LRRK2, NLRP3, EIF2AK2, TRAF2, UNC5C, TNF, LRP6, SNCA |
| GO:0032269~negative regulation of cellular protein metabolic process | 10 | 2.53E-07 | SERPINA1, DAPK1, GSTP1, LRRK2, EIF4EBP1, UBE2D1, EIF2AK2, TNF, LRP6, SNCA |
| GO:0030162~regulation of proteolysis | 9 | 2.89E-07 | GSK3B, SERPINA1, DAPK1, LRRK2, NLRP3, UBE2D1, TRAF2, TNF, SNCA |
| GO:0010033~response to organic substance | 14 | 3.07E-07 | GSK3B, DAPK1, GSTP1, LRRK2, EIF2AK2, UBE2D1, TRAF2, TNF, SLC6A3, SLC6A4, LRP6, EIF4EBP1, NLRP3, SNCA |
| GO:0051248~negative regulation of protein metabolic process | 10 | 4.07E-07 | SERPINA1, DAPK1, GSTP1, LRRK2, EIF4EBP1, UBE2D1, EIF2AK2, TNF, LRP6, SNCA |
| GO:0012501~programmed cell death | 12 | 4.34E-07 | GSK3B, YWHAQ, DAPK1, GSTP1, LRRK2, NLRP3, EIF2AK2, TRAF2, UNC5C, TNF, LRP6, SNCA |
| GO:0032268~regulation of cellular protein metabolic process | 13 | 4.40E-07 | GSK3B, SERPINA1, DAPK1, GSTP1, LRRK2, EIF2AK2, UBE2D1, TRAF2, TNF, LRP6, EIF4EBP1, NLRP3, SNCA |
| GO:0050790~regulation of catalytic activity | 13 | 4.64E-07 | GSK3B, SERPINA1, DAPK1, GSTP1, LRRK2, EIF2AK2, UBE2D1, TRAF2, TNF, LRP6, RCAN1, NLRP3, SNCA |
| GO:0019222~regulation of metabolic process | 18 | 4.72E-07 | GSK3B, SERPINA1, DAPK1, GSTP1, LRRK2, EIF2AK2, UBE2D1, TRAF2, TNF, SLC6A3, SLC6A4, LRP6, RCAN1, YWHAQ, EIF4EBP1, NLRP3, RAB7A, SNCA |
| GO:0044700~single organism signaling | 18 | 5.19E-07 | GSK3B, DAPK1, GSTP1, LRRK2, EIF2AK2, UBE2D1, TRAF2, UNC5C, TNF, SLC6A3, SLC6A4, LRP6, RCAN1, YWHAQ, EIF4EBP1, NLRP3, RAB7A, SNCA |
| GO:0009968~negative regulation of signal transduction | 10 | 5.60E-07 | GSK3B, DAPK1, GSTP1, LRRK2, NLRP3, TRAF2, TNF, RAB7A, LRP6, SNCA |
| GO:0023052~signaling | 18 | 5.97E-07 | GSK3B, DAPK1, GSTP1, LRRK2, EIF2AK2, UBE2D1, TRAF2, UNC5C, TNF, SLC6A3, SLC6A4, LRP6, RCAN1, YWHAQ, EIF4EBP1, NLRP3, RAB7A, SNCA |
| GO:0048583~regulation of response to stimulus | 15 | 6.09E-07 | GSK3B, DAPK1, GSTP1, LRRK2, EIF2AK2, UBE2D1, TRAF2, TNF, SLC6A3, LRP6, RCAN1, YWHAQ, NLRP3, RAB7A, SNCA |
| GO:0007154~cell communication | 18 | 6.21E-07 | GSK3B, DAPK1, GSTP1, LRRK2, EIF2AK2, UBE2D1, TRAF2, UNC5C, TNF, SLC6A3, SLC6A4, LRP6, RCAN1, YWHAQ, EIF4EBP1, NLRP3, RAB7A, SNCA |
| GO:0048518~positive regulation of biological process | 17 | 6.93E-07 | GSK3B, DAPK1, GSTP1, LRRK2, EIF2AK2, UBE2D1, TRAF2, UNC5C, TNF, SLC6A3, SLC6A4, LRP6, YWHAQ, EIF4EBP1, NLRP3, RAB7A, SNCA |
| GO:0010646~regulation of cell communication | 14 | 6.97E-07 | GSK3B, DAPK1, GSTP1, LRRK2, EIF2AK2, TRAF2, TNF, SLC6A4, LRP6, RCAN1, YWHAQ, NLRP3, RAB7A, SNCA |
| GO:0008219~cell death | 12 | 7.67E-07 | GSK3B, YWHAQ, DAPK1, GSTP1, LRRK2, NLRP3, EIF2AK2, TRAF2, UNC5C, TNF, LRP6, SNCA |
| GO:0023051~regulation of signaling | 14 | 8.49E-07 | GSK3B, DAPK1, GSTP1, LRRK2, EIF2AK2, TRAF2, TNF, SLC6A4, LRP6, RCAN1, YWHAQ, NLRP3, RAB7A, SNCA |
| GO:0043066~negative regulation of apoptotic process | 9 | 8.67E-07 | GSK3B, DAPK1, GSTP1, LRRK2, EIF2AK2, TRAF2, TNF, LRP6, SNCA |
| GO:0043069~negative regulation of programmed cell death | 9 | 9.60E-07 | GSK3B, DAPK1, GSTP1, LRRK2, EIF2AK2, TRAF2, TNF, LRP6, SNCA |
| GO:0043065~positive regulation of apoptotic process | 8 | 1.18E-06 | GSK3B, YWHAQ, DAPK1, NLRP3, TRAF2, UNC5C, TNF, SNCA |
| GO:0070887~cellular response to chemical stimulus | 13 | 1.68E-06 | GSK3B, DAPK1, GSTP1, LRRK2, EIF2AK2, UBE2D1, TRAF2, TNF, SLC6A4, LRP6, EIF4EBP1, NLRP3, SNCA |
| GO:1901698~response to nitrogen compound | 9 | 1.71E-06 | GSTP1, LRRK2, EIF4EBP1, TRAF2, TNF, SLC6A3, SLC6A4, LRP6, SNCA |
| GO:0060548~negative regulation of cell death | 9 | 1.74E-06 | GSK3B, DAPK1, GSTP1, LRRK2, EIF2AK2, TRAF2, TNF, LRP6, SNCA |
| GO:0007417~central nervous system development | 9 | 1.85E-06 | RCAN1, GSK3B, YWHAQ, GSTP1, LRRK2, UNC5C, SLC6A3, SLC6A4, LRP6 |
| GO:0048522~positive regulation of cellular process | 16 | 1.87E-06 | GSK3B, DAPK1, GSTP1, LRRK2, EIF2AK2, UBE2D1, TRAF2, UNC5C, TNF, SLC6A4, LRP6, YWHAQ, EIF4EBP1, NLRP3, RAB7A, SNCA |
| GO:2001233~regulation of apoptotic signaling pathway | 7 | 1.90E-06 | GSK3B, YWHAQ, DAPK1, GSTP1, LRRK2, TRAF2, TNF |
| GO:0071822~protein complex subunit organization | 11 | 1.95E-06 | GSK3B, SERPINA1, LRRK2, EIF4EBP1, NLRP3, EIF2AK2, TRAF2, TNF, SLC6A4, LRP6, SNCA |
| GO:1901701~cellular response to oxygen-containing compound | 9 | 1.97E-06 | GSTP1, LRRK2, EIF4EBP1, NLRP3, TRAF2, TNF, SLC6A4, LRP6, SNCA |
| GO:0009966~regulation of signal transduction | 13 | 2.33E-06 | GSK3B, DAPK1, GSTP1, LRRK2, EIF2AK2, TRAF2, TNF, LRP6, RCAN1, YWHAQ, NLRP3, RAB7A, SNCA |
| GO:0051338~regulation of transferase activity | 9 | 2.79E-06 | GSK3B, GSTP1, LRRK2, UBE2D1, EIF2AK2, TRAF2, TNF, LRP6, SNCA |
| GO:0080090~regulation of primary metabolic process | 17 | 2.81E-06 | GSK3B, SERPINA1, DAPK1, GSTP1, LRRK2, EIF2AK2, UBE2D1, TRAF2, TNF, SLC6A3, LRP6, RCAN1, YWHAQ, EIF4EBP1, NLRP3, RAB7A, SNCA |
| GO:0060255~regulation of macromolecule metabolic process | 17 | 2.94E-06 | GSK3B, SERPINA1, DAPK1, GSTP1, LRRK2, EIF2AK2, UBE2D1, TRAF2, TNF, SLC6A4, LRP6, RCAN1, YWHAQ, EIF4EBP1, NLRP3, RAB7A, SNCA |
| GO:0042221~response to chemical | 15 | 3.03E-06 | GSK3B, DAPK1, GSTP1, LRRK2, EIF2AK2, UBE2D1, TRAF2, UNC5C, TNF, SLC6A3, SLC6A4, LRP6, EIF4EBP1, NLRP3, SNCA |
| GO:0050896~response to stimulus | 19 | 3.27E-06 | GSK3B, SERPINA1, DAPK1, GSTP1, LRRK2, EIF2AK2, UBE2D1, TRAF2, UNC5C, TNF, SLC6A3, SLC6A4, LRP6, RCAN1, YWHAQ, EIF4EBP1, NLRP3, RAB7A, SNCA |
| GO:0065009~regulation of molecular function | 13 | 3.96E-06 | GSK3B, SERPINA1, DAPK1, GSTP1, LRRK2, EIF2AK2, UBE2D1, TRAF2, TNF, LRP6, RCAN1, NLRP3, SNCA |
| GO:0044707~single-multicellular organism process | 17 | 4.08E-06 | GSK3B, SERPINA1, GSTP1, LRRK2, EIF2AK2, TRAF2, UNC5C, TNF, SLC6A3, SLC6A4, LRP6, RCAN1, YWHAQ, EIF4EBP1, NLRP3, RAB7A, SNCA |
| GO:0010605~negative regulation of macromolecule metabolic process | 12 | 4.37E-06 | GSK3B, SERPINA1, YWHAQ, DAPK1, GSTP1, LRRK2, EIF4EBP1, UBE2D1, EIF2AK2, TNF, LRP6, SNCA |
| GO:0031324~negative regulation of cellular metabolic process | 12 | 4.74E-06 | GSK3B, SERPINA1, YWHAQ, DAPK1, GSTP1, LRRK2, EIF4EBP1, UBE2D1, EIF2AK2, TNF, LRP6, SNCA |
| GO:0051051~negative regulation of transport | 7 | 5.06E-06 | GSK3B, YWHAQ, LRRK2, NLRP3, TNF, RAB7A, SNCA |
| GO:0006461~protein complex assembly | 10 | 5.99E-06 | GSK3B, SERPINA1, LRRK2, EIF4EBP1, NLRP3, EIF2AK2, TRAF2, TNF, SLC6A4, SNCA |
| GO:0070271~protein complex biogenesis | 10 | 6.03E-06 | GSK3B, SERPINA1, LRRK2, EIF4EBP1, NLRP3, EIF2AK2, TRAF2, TNF, SLC6A4, SNCA |
| GO:1901700~response to oxygen-containing compound | 10 | 6.75E-06 | GSTP1, LRRK2, EIF4EBP1, NLRP3, TRAF2, TNF, SLC6A3, SLC6A4, LRP6, SNCA |
| GO:0043280~positive regulation of cysteine-type endopeptidase activity involved in apoptotic process | 5 | 6.79E-06 | DAPK1, NLRP3, TRAF2, TNF, SNCA |
| GO:0071900~regulation of protein serine/threonine kinase activity | 7 | 6.98E-06 | GSTP1, LRRK2, EIF2AK2, TRAF2, TNF, LRP6, SNCA |
| GO:0009893~positive regulation of metabolic process | 13 | 7.93E-06 | GSK3B, DAPK1, GSTP1, LRRK2, EIF2AK2, UBE2D1, TRAF2, TNF, SLC6A4, LRP6, NLRP3, RAB7A, SNCA |
| GO:0044092~negative regulation of molecular function | 9 | 8.00E-06 | GSK3B, SERPINA1, GSTP1, LRRK2, NLRP3, UBE2D1, TNF, LRP6, SNCA |
| GO:0051247~positive regulation of protein metabolic process | 10 | 8.52E-06 | GSK3B, DAPK1, LRRK2, NLRP3, UBE2D1, EIF2AK2, TRAF2, TNF, RAB7A, SNCA |
| GO:2001056~positive regulation of cysteine-type endopeptidase activity | 5 | 9.07E-06 | DAPK1, NLRP3, TRAF2, TNF, SNCA |
| GO:0009892~negative regulation of metabolic process | 12 | 9.79E-06 | GSK3B, SERPINA1, YWHAQ, DAPK1, GSTP1, LRRK2, EIF4EBP1, UBE2D1, EIF2AK2, TNF, LRP6, SNCA |
| GO:0010243~response to organonitrogen compound | 8 | 9.94E-06 | GSTP1, LRRK2, EIF4EBP1, TNF, SLC6A3, SLC6A4, LRP6, SNCA |
| GO:0051649~establishment of localization in cell | 11 | 1.01E-05 | GSK3B, SERPINA1, YWHAQ, LRRK2, NLRP3, TNF, SLC6A3, SLC6A4, RAB7A, LRP6, SNCA |
| GO:0048513~animal organ development | 13 | 1.04E-05 | GSK3B, GSTP1, LRRK2, EIF2AK2, UNC5C, TNF, SLC6A3, SLC6A4, LRP6, RCAN1, YWHAQ, EIF4EBP1, NLRP3 |
| GO:0071407~cellular response to organic cyclic compound | 7 | 1.16E-05 | GSTP1, LRRK2, EIF4EBP1, TNF, SLC6A4, LRP6, SNCA |
| GO:0010950~positive regulation of endopeptidase activity | 5 | 1.33E-05 | DAPK1, NLRP3, TRAF2, TNF, SNCA |
| GO:0009605~response to external stimulus | 11 | 1.37E-05 | RCAN1, GSTP1, LRRK2, EIF4EBP1, NLRP3, EIF2AK2, UNC5C, TNF, SLC6A3, SLC6A4, SNCA |
| GO:0043086~negative regulation of catalytic activity | 8 | 1.49E-05 | GSK3B, SERPINA1, GSTP1, LRRK2, UBE2D1, TNF, LRP6, SNCA |
| GO:0035556~intracellular signal transduction | 12 | 1.64E-05 | RCAN1, GSK3B, YWHAQ, DAPK1, GSTP1, LRRK2, EIF4EBP1, NLRP3, EIF2AK2, TRAF2, TNF, RAB7A |
| GO:0007166~cell surface receptor signaling pathway | 12 | 1.71E-05 | GSK3B, DAPK1, GSTP1, LRRK2, EIF4EBP1, UBE2D1, TRAF2, UNC5C, TNF, RAB7A, LRP6, SNCA |
| GO:1901699~cellular response to nitrogen compound | 7 | 1.73E-05 | GSTP1, LRRK2, EIF4EBP1, TRAF2, TNF, SLC6A4, SNCA |
| GO:0010952~positive regulation of peptidase activity | 5 | 1.93E-05 | DAPK1, NLRP3, TRAF2, TNF, SNCA |
| GO:0009896~positive regulation of catabolic process | 6 | 2.06E-05 | GSK3B, LRRK2, UBE2D1, TNF, RAB7A, SNCA |
| GO:0097190~apoptotic signaling pathway | 7 | 2.22E-05 | GSK3B, YWHAQ, DAPK1, GSTP1, LRRK2, TRAF2, TNF |
| GO:0065003~macromolecular complex assembly | 10 | 2.38E-05 | GSK3B, SERPINA1, LRRK2, EIF4EBP1, NLRP3, EIF2AK2, TRAF2, TNF, SLC6A4, SNCA |
| GO:0014070~response to organic cyclic compound | 8 | 2.45E-05 | GSTP1, LRRK2, EIF4EBP1, TNF, SLC6A3, SLC6A4, LRP6, SNCA |
| GO:0044765~single-organism transport | 13 | 2.65E-05 | GSK3B, SERPINA1, DAPK1, LRRK2, TRAF2, TNF, SLC6A3, SLC6A4, LRP6, YWHAQ, NLRP3, RAB7A, SNCA |
| GO:0007165~signal transduction | 16 | 2.81E-05 | GSK3B, DAPK1, GSTP1, LRRK2, EIF2AK2, UBE2D1, TRAF2, UNC5C, TNF, LRP6, RCAN1, YWHAQ, EIF4EBP1, NLRP3, RAB7A, SNCA |
| GO:0031323~regulation of cellular metabolic process | 16 | 3.30E-05 | GSK3B, SERPINA1, DAPK1, GSTP1, LRRK2, EIF2AK2, UBE2D1, TRAF2, TNF, SLC6A3, LRP6, RCAN1, YWHAQ, EIF4EBP1, NLRP3, SNCA |
| GO:0010604~positive regulation of macromolecule metabolic process | 12 | 3.32E-05 | GSK3B, DAPK1, LRRK2, NLRP3, UBE2D1, EIF2AK2, TRAF2, TNF, SLC6A4, RAB7A, LRP6, SNCA |
| GO:0052548~regulation of endopeptidase activity | 6 | 3.51E-05 | SERPINA1, DAPK1, NLRP3, TRAF2, TNF, SNCA |
| GO:0051716~cellular response to stimulus | 17 | 4.00E-05 | GSK3B, DAPK1, GSTP1, LRRK2, EIF2AK2, UBE2D1, TRAF2, UNC5C, TNF, SLC6A4, LRP6, RCAN1, YWHAQ, EIF4EBP1, NLRP3, RAB7A, SNCA |
| GO:0044093~positive regulation of molecular function | 10 | 4.31E-05 | GSK3B, DAPK1, LRRK2, NLRP3, UBE2D1, EIF2AK2, TRAF2, TNF, LRP6, SNCA |
| GO:0052547~regulation of peptidase activity | 6 | 4.73E-05 | SERPINA1, DAPK1, NLRP3, TRAF2, TNF, SNCA |
| GO:1902578~single-organism localization | 13 | 4.83E-05 | GSK3B, SERPINA1, DAPK1, LRRK2, TRAF2, TNF, SLC6A3, SLC6A4, LRP6, YWHAQ, NLRP3, RAB7A, SNCA |
| GO:0032501~multicellular organismal process | 17 | 4.94E-05 | GSK3B, SERPINA1, GSTP1, LRRK2, EIF2AK2, TRAF2, UNC5C, TNF, SLC6A3, SLC6A4, LRP6, RCAN1, YWHAQ, EIF4EBP1, NLRP3, RAB7A, SNCA |
| GO:0010468~regulation of gene expression | 14 | 5.03E-05 | GSK3B, DAPK1, LRRK2, EIF2AK2, UBE2D1, TRAF2, TNF, SLC6A4, LRP6, RCAN1, YWHAQ, EIF4EBP1, NLRP3, SNCA |
| GO:0010558~negative regulation of macromolecule biosynthetic process | 9 | 5.13E-05 | GSK3B, YWHAQ, DAPK1, GSTP1, EIF4EBP1, UBE2D1, EIF2AK2, TNF, SNCA |
| GO:0032270~positive regulation of cellular protein metabolic process | 9 | 5.18E-05 | GSK3B, DAPK1, LRRK2, NLRP3, UBE2D1, EIF2AK2, TRAF2, TNF, SNCA |
| GO:0043281~regulation of cysteine-type endopeptidase activity involved in apoptotic process | 5 | 5.24E-05 | DAPK1, NLRP3, TRAF2, TNF, SNCA |
| GO:0007420~brain development | 7 | 5.45E-05 | GSK3B, YWHAQ, LRRK2, UNC5C, SLC6A3, SLC6A4, LRP6 |
| GO:0006950~response to stress | 13 | 5.79E-05 | GSK3B, SERPINA1, DAPK1, GSTP1, LRRK2, EIF2AK2, UBE2D1, TRAF2, TNF, SLC6A4, EIF4EBP1, NLRP3, SNCA |
| GO:0043933~macromolecular complex subunit organization | 11 | 6.34E-05 | GSK3B, SERPINA1, LRRK2, EIF4EBP1, NLRP3, EIF2AK2, TRAF2, TNF, SLC6A4, LRP6, SNCA |
| GO:2001234~negative regulation of apoptotic signaling pathway | 5 | 6.34E-05 | DAPK1, GSTP1, LRRK2, TRAF2, TNF |
| GO:0032879~regulation of localization | 11 | 6.61E-05 | GSK3B, YWHAQ, DAPK1, GSTP1, LRRK2, NLRP3, TRAF2, UNC5C, TNF, RAB7A, SNCA |
| GO:0060322~head development | 7 | 7.25E-05 | GSK3B, YWHAQ, LRRK2, UNC5C, SLC6A3, SLC6A4, LRP6 |
| GO:0045859~regulation of protein kinase activity | 7 | 7.83E-05 | GSTP1, LRRK2, EIF2AK2, TRAF2, TNF, LRP6, SNCA |
| GO:0009890~negative regulation of biosynthetic process | 9 | 7.84E-05 | GSK3B, YWHAQ, DAPK1, GSTP1, EIF4EBP1, UBE2D1, EIF2AK2, TNF, SNCA |
| GO:2000116~regulation of cysteine-type endopeptidase activity | 5 | 8.14E-05 | DAPK1, NLRP3, TRAF2, TNF, SNCA |
| GO:0097191~extrinsic apoptotic signaling pathway | 5 | 8.29E-05 | GSK3B, DAPK1, GSTP1, TRAF2, TNF |
| GO:0051641~cellular localization | 11 | 8.39E-05 | GSK3B, SERPINA1, YWHAQ, LRRK2, NLRP3, TNF, SLC6A3, SLC6A4, RAB7A, LRP6, SNCA |
| GO:0046903~secretion | 8 | 8.42E-05 | SERPINA1, LRRK2, NLRP3, TRAF2, TNF, SLC6A3, RAB7A, SNCA |
| GO:0048584~positive regulation of response to stimulus | 10 | 9.45E-05 | GSK3B, YWHAQ, LRRK2, NLRP3, UBE2D1, EIF2AK2, TRAF2, TNF, LRP6, SNCA |
| GO:0051091~positive regulation of sequence-specific DNA binding transcription factor activity | 5 | 9.49E-05 | NLRP3, EIF2AK2, TRAF2, TNF, LRP6 |
| GO:0009719~response to endogenous stimulus | 9 | 9.63E-05 | GSTP1, LRRK2, EIF4EBP1, UBE2D1, TNF, SLC6A3, SLC6A4, LRP6, SNCA |

**Table S11.** Biological processes related to seventh group of genes (Cluster 7)

| **Term** | **Count** | **PValue** | **Genes** |
| --- | --- | --- | --- |
| GO:0007169~transmembrane receptor protein tyrosine kinase signaling pathway | 11 | 4.21E-13 | NTRK1, NTRK2, NGFR, BDNF, IRS1, LCK, NTF3, PTK2B, PIK3R1, NGF, SORCS3 |
| GO:0007167~enzyme linked receptor protein signaling pathway | 11 | 1.99E-11 | NTRK1, NTRK2, NGFR, BDNF, IRS1, LCK, NTF3, PTK2B, PIK3R1, NGF, SORCS3 |
| GO:0007166~cell surface receptor signaling pathway | 13 | 2.55E-10 | NTRK1, NGFR, NTRK2, BDNF, IRS1, NEDD9, PIK3R1, NGF, SORCS3, LCK, NTF3, PTK2B, CD44 |
| GO:0009967~positive regulation of signal transduction | 11 | 1.08E-09 | NTRK1, NTRK2, NGFR, BDNF, IRS1, LCK, NTF3, PTK2B, PIK3R1, NGF, CD44 |
| GO:0010647~positive regulation of cell communication | 11 | 2.50E-09 | NTRK1, NTRK2, NGFR, BDNF, IRS1, LCK, NTF3, PTK2B, PIK3R1, NGF, CD44 |
| GO:0023056~positive regulation of signaling | 11 | 2.63E-09 | NTRK1, NTRK2, NGFR, BDNF, IRS1, LCK, NTF3, PTK2B, PIK3R1, NGF, CD44 |
| GO:0038179~neurotrophin signaling pathway | 5 | 3.52E-09 | NTRK1, NTRK2, NGFR, NGF, SORCS3 |
| GO:0043066~negative regulation of apoptotic process | 9 | 1.28E-08 | NTRK1, NTRK2, NGFR, BDNF, NTF3, PTK2B, PIK3R1, NGF, CD44 |
| GO:0043069~negative regulation of programmed cell death | 9 | 1.42E-08 | NTRK1, NTRK2, NGFR, BDNF, NTF3, PTK2B, PIK3R1, NGF, CD44 |
| GO:0060548~negative regulation of cell death | 9 | 2.63E-08 | NTRK1, NTRK2, NGFR, BDNF, NTF3, PTK2B, PIK3R1, NGF, CD44 |
| GO:0043524~negative regulation of neuron apoptotic process | 6 | 2.64E-08 | NTRK1, NTRK2, BDNF, NTF3, PTK2B, NGF |
| GO:0042981~regulation of apoptotic process | 10 | 3.31E-08 | NTRK1, NTRK2, NGFR, BDNF, LCK, NTF3, PTK2B, PIK3R1, NGF, CD44 |
| GO:0048584~positive regulation of response to stimulus | 11 | 3.42E-08 | NTRK1, NTRK2, NGFR, BDNF, IRS1, LCK, NTF3, PTK2B, PIK3R1, NGF, CD44 |
| GO:0043067~regulation of programmed cell death | 10 | 3.60E-08 | NTRK1, NTRK2, NGFR, BDNF, LCK, NTF3, PTK2B, PIK3R1, NGF, CD44 |
| GO:0010646~regulation of cell communication | 12 | 6.29E-08 | NTRK1, NTRK2, NGFR, BDNF, IRS1, LCK, NTF3, PTK2B, PIK3R1, NGF, SORCS3, CD44 |
| GO:0010941~regulation of cell death | 10 | 6.40E-08 | NTRK1, NTRK2, NGFR, BDNF, LCK, NTF3, PTK2B, PIK3R1, NGF, CD44 |
| GO:0023051~regulation of signaling | 12 | 7.51E-08 | NTRK1, NTRK2, NGFR, BDNF, IRS1, LCK, NTF3, PTK2B, PIK3R1, NGF, SORCS3, CD44 |
| GO:1901215~negative regulation of neuron death | 6 | 8.92E-08 | NTRK1, NTRK2, BDNF, NTF3, PTK2B, NGF |
| GO:0048015~phosphatidylinositol-mediated signaling | 6 | 1.11E-07 | NTRK1, NTRK2, IRS1, LCK, PIK3R1, NGF |
| GO:0048017~inositol lipid-mediated signaling | 6 | 1.21E-07 | NTRK1, NTRK2, IRS1, LCK, PIK3R1, NGF |
| GO:0043523~regulation of neuron apoptotic process | 6 | 1.41E-07 | NTRK1, NTRK2, BDNF, NTF3, PTK2B, NGF |
| GO:0051402~neuron apoptotic process | 6 | 1.86E-07 | NTRK1, NTRK2, BDNF, NTF3, PTK2B, NGF |
| GO:1902531~regulation of intracellular signal transduction | 10 | 1.95E-07 | NTRK1, NTRK2, NGFR, IRS1, LCK, NTF3, PTK2B, PIK3R1, NGF, CD44 |
| GO:0006915~apoptotic process | 10 | 2.30E-07 | NTRK1, NTRK2, NGFR, BDNF, LCK, NTF3, PTK2B, PIK3R1, NGF, CD44 |
| GO:0006928~movement of cell or subcellular component | 10 | 2.91E-07 | NTRK1, NTRK2, NGFR, BDNF, LCK, NTF3, PTK2B, PIK3R1, NGF, CD44 |
| GO:0010976~positive regulation of neuron projection development | 6 | 3.31E-07 | NTRK1, NTRK2, NGFR, BDNF, PTK2B, NGF |
| GO:0048011~neurotrophin TRK receptor signaling pathway | 4 | 3.68E-07 | NTRK1, NGFR, NGF, SORCS3 |
| GO:0012501~programmed cell death | 10 | 3.82E-07 | NTRK1, NTRK2, NGFR, BDNF, LCK, NTF3, PTK2B, PIK3R1, NGF, CD44 |
| GO:0006468~protein phosphorylation | 10 | 3.82E-07 | NTRK1, NTRK2, NGFR, IRS1, LCK, NTF3, PTK2B, PIK3R1, NGF, CD44 |
| GO:0043410~positive regulation of MAPK cascade | 7 | 4.49E-07 | NTRK1, NTRK2, NGFR, NTF3, PTK2B, NGF, CD44 |
| GO:0000165~MAPK cascade | 8 | 4.90E-07 | NTRK1, NTRK2, NGFR, IRS1, NTF3, PTK2B, NGF, CD44 |
| GO:0051336~regulation of hydrolase activity | 9 | 5.86E-07 | NTRK1, NTRK2, NGFR, IRS1, LCK, NTF3, PTK2B, NGF, CD44 |
| GO:1901214~regulation of neuron death | 6 | 5.86E-07 | NTRK1, NTRK2, BDNF, NTF3, PTK2B, NGF |
| GO:0009966~regulation of signal transduction | 11 | 6.06E-07 | NTRK1, NTRK2, NGFR, BDNF, IRS1, LCK, NTF3, PTK2B, PIK3R1, NGF, CD44 |
| GO:0008219~cell death | 10 | 6.20E-07 | NTRK1, NTRK2, NGFR, BDNF, LCK, NTF3, PTK2B, PIK3R1, NGF, CD44 |
| GO:0023014~signal transduction by protein phosphorylation | 8 | 6.51E-07 | NTRK1, NTRK2, NGFR, IRS1, NTF3, PTK2B, NGF, CD44 |
| GO:0042325~regulation of phosphorylation | 9 | 8.04E-07 | NTRK1, NTRK2, NGFR, IRS1, NTF3, PTK2B, PIK3R1, NGF, CD44 |
| GO:0070997~neuron death | 6 | 8.49E-07 | NTRK1, NTRK2, BDNF, NTF3, PTK2B, NGF |
| GO:1902533~positive regulation of intracellular signal transduction | 8 | 8.68E-07 | NTRK1, NTRK2, NGFR, LCK, NTF3, PTK2B, NGF, CD44 |
| GO:0065009~regulation of molecular function | 11 | 9.67E-07 | NTRK1, NTRK2, NGFR, BDNF, IRS1, LCK, NTF3, PTK2B, PIK3R1, NGF, CD44 |
| GO:0042327~positive regulation of phosphorylation | 8 | 9.83E-07 | NTRK1, NTRK2, NGFR, IRS1, NTF3, PTK2B, NGF, CD44 |
| GO:0045664~regulation of neuron differentiation | 7 | 1.04E-06 | NTRK1, NTRK2, NGFR, BDNF, NTF3, PTK2B, NGF |
| GO:0031346~positive regulation of cell projection organization | 6 | 1.34E-06 | NTRK1, NTRK2, NGFR, BDNF, PTK2B, NGF |
| GO:0045666~positive regulation of neuron differentiation | 6 | 1.48E-06 | NTRK1, NTRK2, NGFR, BDNF, PTK2B, NGF |
| GO:0040011~locomotion | 9 | 1.65E-06 | NTRK1, NTRK2, NGFR, BDNF, LCK, NTF3, PTK2B, PIK3R1, CD44 |
| GO:0042221~response to chemical | 12 | 1.77E-06 | NTRK1, NTRK2, NGFR, BDNF, IRS1, LCK, NTF3, PTK2B, PIK3R1, NGF, SORCS3, CD44 |
| GO:0016310~phosphorylation | 10 | 1.90E-06 | NTRK1, NTRK2, NGFR, IRS1, LCK, NTF3, PTK2B, PIK3R1, NGF, CD44 |
| GO:0010562~positive regulation of phosphorus metabolic process | 8 | 2.18E-06 | NTRK1, NTRK2, NGFR, IRS1, NTF3, PTK2B, NGF, CD44 |
| GO:0045937~positive regulation of phosphate metabolic process | 8 | 2.18E-06 | NTRK1, NTRK2, NGFR, IRS1, NTF3, PTK2B, NGF, CD44 |
| GO:0019220~regulation of phosphate metabolic process | 9 | 2.44E-06 | NTRK1, NTRK2, NGFR, IRS1, NTF3, PTK2B, PIK3R1, NGF, CD44 |
| GO:0051174~regulation of phosphorus metabolic process | 9 | 2.45E-06 | NTRK1, NTRK2, NGFR, IRS1, NTF3, PTK2B, PIK3R1, NGF, CD44 |
| GO:0018108~peptidyl-tyrosine phosphorylation | 6 | 2.65E-06 | NTRK1, NTRK2, LCK, NTF3, PTK2B, CD44 |
| GO:0007165~signal transduction | 13 | 2.72E-06 | NTRK1, NGFR, NTRK2, BDNF, IRS1, NEDD9, PIK3R1, NGF, SORCS3, LCK, NTF3, PTK2B, CD44 |
| GO:0018212~peptidyl-tyrosine modification | 6 | 2.72E-06 | NTRK1, NTRK2, LCK, NTF3, PTK2B, CD44 |
| GO:0050790~regulation of catalytic activity | 10 | 3.07E-06 | NTRK1, NTRK2, NGFR, IRS1, LCK, NTF3, PTK2B, PIK3R1, NGF, CD44 |
| GO:0043408~regulation of MAPK cascade | 7 | 3.18E-06 | NTRK1, NTRK2, NGFR, NTF3, PTK2B, NGF, CD44 |
| GO:0050767~regulation of neurogenesis | 7 | 3.29E-06 | NTRK1, NTRK2, NGFR, BDNF, NTF3, PTK2B, NGF |
| GO:0050769~positive regulation of neurogenesis | 6 | 4.39E-06 | NTRK1, NTRK2, NGFR, BDNF, PTK2B, NGF |
| GO:0051130~positive regulation of cellular component organization | 8 | 4.89E-06 | NTRK1, NTRK2, NGFR, BDNF, NTF3, PTK2B, PIK3R1, NGF |
| GO:0010975~regulation of neuron projection development | 6 | 6.03E-06 | NTRK1, NTRK2, NGFR, BDNF, PTK2B, NGF |
| GO:0044700~single organism signaling | 13 | 6.46E-06 | NTRK1, NGFR, NTRK2, BDNF, IRS1, NEDD9, PIK3R1, NGF, SORCS3, LCK, NTF3, PTK2B, CD44 |
| GO:0051960~regulation of nervous system development | 7 | 6.59E-06 | NTRK1, NTRK2, NGFR, BDNF, NTF3, PTK2B, NGF |
| GO:0044093~positive regulation of molecular function | 9 | 6.75E-06 | NTRK1, NGFR, BDNF, IRS1, LCK, NTF3, PTK2B, NGF, CD44 |
| GO:0023052~signaling | 13 | 7.16E-06 | NTRK1, NGFR, NTRK2, BDNF, IRS1, NEDD9, PIK3R1, NGF, SORCS3, LCK, NTF3, PTK2B, CD44 |
| GO:0007154~cell communication | 13 | 7.37E-06 | NTRK1, NGFR, NTRK2, BDNF, IRS1, NEDD9, PIK3R1, NGF, SORCS3, LCK, NTF3, PTK2B, CD44 |
| GO:0043549~regulation of kinase activity | 7 | 7.59E-06 | NTRK1, IRS1, NTF3, PTK2B, PIK3R1, NGF, CD44 |
| GO:0035556~intracellular signal transduction | 10 | 8.55E-06 | NTRK1, NTRK2, NGFR, IRS1, LCK, NTF3, PTK2B, PIK3R1, NGF, CD44 |
| GO:0051962~positive regulation of nervous system development | 6 | 9.06E-06 | NTRK1, NTRK2, NGFR, BDNF, PTK2B, NGF |
| GO:0031175~neuron projection development | 7 | 9.35E-06 | NTRK1, NTRK2, NGFR, BDNF, NTF3, PTK2B, NGF |
| GO:0048583~regulation of response to stimulus | 11 | 9.57E-06 | NTRK1, NTRK2, NGFR, BDNF, IRS1, LCK, NTF3, PTK2B, PIK3R1, NGF, CD44 |
| GO:0060284~regulation of cell development | 7 | 1.18E-05 | NTRK1, NTRK2, NGFR, BDNF, NTF3, PTK2B, NGF |
| GO:0010720~positive regulation of cell development | 6 | 1.29E-05 | NTRK1, NTRK2, NGFR, BDNF, PTK2B, NGF |
| GO:0050772~positive regulation of axonogenesis | 4 | 1.42E-05 | NTRK2, NGFR, BDNF, NGF |
| GO:0031325~positive regulation of cellular metabolic process | 10 | 1.50E-05 | NTRK1, NTRK2, NGFR, IRS1, LCK, NTF3, PTK2B, PIK3R1, NGF, CD44 |
| GO:0001934~positive regulation of protein phosphorylation | 7 | 1.53E-05 | NTRK1, NTRK2, NGFR, NTF3, PTK2B, NGF, CD44 |
| GO:0010604~positive regulation of macromolecule metabolic process | 10 | 1.58E-05 | NTRK1, NTRK2, NGFR, IRS1, LCK, NTF3, PTK2B, PIK3R1, NGF, CD44 |
| GO:0033674~positive regulation of kinase activity | 6 | 1.60E-05 | NTRK1, IRS1, NTF3, PTK2B, NGF, CD44 |
| GO:0032270~positive regulation of cellular protein metabolic process | 8 | 1.65E-05 | NTRK1, NTRK2, NGFR, LCK, NTF3, PTK2B, NGF, CD44 |
| GO:0050803~regulation of synapse structure or activity | 5 | 1.75E-05 | NTRK1, NTRK2, BDNF, PTK2B, SORCS3 |
| GO:0051345~positive regulation of hydrolase activity | 7 | 1.81E-05 | NTRK1, NGFR, IRS1, LCK, NTF3, PTK2B, NGF |
| GO:0048812~neuron projection morphogenesis | 6 | 2.28E-05 | NTRK1, NTRK2, NGFR, BDNF, NTF3, NGF |
| GO:0051338~regulation of transferase activity | 7 | 2.29E-05 | NTRK1, IRS1, NTF3, PTK2B, PIK3R1, NGF, CD44 |
| GO:0051716~cellular response to stimulus | 13 | 2.41E-05 | NTRK1, NGFR, NTRK2, BDNF, IRS1, NEDD9, PIK3R1, NGF, SORCS3, LCK, NTF3, PTK2B, CD44 |
| GO:0048666~neuron development | 7 | 2.43E-05 | NTRK1, NTRK2, NGFR, BDNF, NTF3, PTK2B, NGF |
| GO:0051247~positive regulation of protein metabolic process | 8 | 2.45E-05 | NTRK1, NTRK2, NGFR, LCK, NTF3, PTK2B, NGF, CD44 |
| GO:0071310~cellular response to organic substance | 9 | 2.48E-05 | NTRK1, NTRK2, NGFR, IRS1, PTK2B, PIK3R1, NGF, SORCS3, CD44 |
| GO:0007267~cell-cell signaling | 8 | 2.55E-05 | NTRK1, NTRK2, BDNF, IRS1, NTF3, PTK2B, NGF, SORCS3 |
| GO:0009893~positive regulation of metabolic process | 10 | 2.77E-05 | NTRK1, NTRK2, NGFR, IRS1, LCK, NTF3, PTK2B, PIK3R1, NGF, CD44 |
| GO:0045595~regulation of cell differentiation | 8 | 2.87E-05 | NTRK1, NTRK2, NGFR, BDNF, NTF3, PTK2B, PIK3R1, NGF |
| GO:0031344~regulation of cell projection organization | 6 | 2.93E-05 | NTRK1, NTRK2, NGFR, BDNF, PTK2B, NGF |
| GO:0006796~phosphate-containing compound metabolic process | 10 | 3.01E-05 | NTRK1, NTRK2, NGFR, IRS1, LCK, NTF3, PTK2B, PIK3R1, NGF, CD44 |
| GO:0043085~positive regulation of catalytic activity | 8 | 3.05E-05 | NTRK1, NGFR, IRS1, LCK, NTF3, PTK2B, NGF, CD44 |
| GO:0006793~phosphorus metabolic process | 10 | 3.06E-05 | NTRK1, NTRK2, NGFR, IRS1, LCK, NTF3, PTK2B, PIK3R1, NGF, CD44 |
| GO:0071363~cellular response to growth factor stimulus | 6 | 3.99E-05 | NTRK1, NTRK2, NGFR, NGF, SORCS3, CD44 |
| GO:0048523~negative regulation of cellular process | 11 | 4.68E-05 | NTRK1, NTRK2, NGFR, BDNF, IRS1, LCK, NTF3, PTK2B, PIK3R1, NGF, CD44 |
| GO:0070848~response to growth factor | 6 | 4.80E-05 | NTRK1, NTRK2, NGFR, NGF, SORCS3, CD44 |
| GO:0051347~positive regulation of transferase activity | 6 | 5.02E-05 | NTRK1, IRS1, NTF3, PTK2B, NGF, CD44 |
| GO:2000026~regulation of multicellular organismal development | 8 | 5.84E-05 | NTRK1, NTRK2, NGFR, BDNF, NTF3, PTK2B, PIK3R1, NGF |
| GO:0031401~positive regulation of protein modification process | 7 | 6.35E-05 | NTRK1, NTRK2, NGFR, NTF3, PTK2B, NGF, CD44 |
| GO:2001273~regulation of glucose import in response to insulin stimulus | 3 | 7.12E-05 | NGFR, IRS1, PIK3R1 |
| GO:0048519~negative regulation of biological process | 11 | 9.23E-05 | NTRK1, NTRK2, NGFR, BDNF, IRS1, LCK, NTF3, PTK2B, PIK3R1, NGF, CD44 |
| GO:0030182~neuron differentiation | 7 | 9.42E-05 | NTRK1, NTRK2, NGFR, BDNF, NTF3, PTK2B, NGF |
| GO:0065008~regulation of biological quality | 10 | 9.76E-05 | NTRK1, NTRK2, NGFR, BDNF, IRS1, LCK, PTK2B, PIK3R1, NGF, SORCS3 |

**Table S12.** Biological processes related to the eighth group of genes (Cluster 8)

| **Term** | **Count** | **PValue** | **Genes** |
| --- | --- | --- | --- |
| GO:0016192~vesicle-mediated transport | 10 | 5.86E-08 | LRPAP1, LRP1, IL1B, LRP2, VLDLR, A2M, CLU, LRP8, LDLR, SORL1 |
| GO:0006897~endocytosis | 8 | 9.86E-08 | LRPAP1, LRP1, IL1B, LRP2, VLDLR, LRP8, LDLR, SORL1 |
| GO:0006810~transport | 13 | 2.08E-07 | IL10, LRPAP1, LRP1, LRP2, VLDLR, CLU, LRP8, SORL1, RELN, ATP7B, IL1B, A2M, LDLR |
| GO:0051234~establishment of localization | 13 | 2.87E-07 | IL10, LRPAP1, LRP1, LRP2, VLDLR, CLU, LRP8, SORL1, RELN, ATP7B, IL1B, A2M, LDLR |
| GO:0006869~lipid transport | 6 | 1.53E-06 | LRP1, IL1B, VLDLR, CLU, LDLR, SORL1 |
| GO:0006898~receptor-mediated endocytosis | 6 | 2.17E-06 | LRP1, LRP2, VLDLR, LRP8, LDLR, SORL1 |
| GO:0010876~lipid localization | 6 | 2.40E-06 | LRP1, IL1B, VLDLR, CLU, LDLR, SORL1 |
| GO:0038026~reelin-mediated signaling pathway | 3 | 2.81E-06 | RELN, VLDLR, LRP8 |
| GO:0051179~localization | 13 | 3.32E-06 | IL10, LRPAP1, LRP1, LRP2, VLDLR, CLU, LRP8, SORL1, RELN, ATP7B, IL1B, A2M, LDLR |
| GO:0044707~single-multicellular organism process | 13 | 3.83E-06 | IL10, LRPAP1, LRP1, LRP2, VLDLR, CLU, LRP8, SORL1, RELN, ATP7B, IL1B, A2M, LDLR |
| GO:0044765~single-organism transport | 11 | 5.26E-06 | IL10, RELN, ATP7B, LRP1, IL1B, LRP2, VLDLR, A2M, CLU, LDLR, SORL1 |
| GO:1902578~single-organism localization | 11 | 9.03E-06 | IL10, RELN, ATP7B, LRP1, IL1B, LRP2, VLDLR, A2M, CLU, LDLR, SORL1 |
| GO:0006629~lipid metabolic process | 8 | 1.07E-05 | LRP1, IL1B, LRP2, VLDLR, CLU, LRP8, LDLR, SORL1 |
| GO:0065009~regulation of molecular function | 10 | 1.61E-05 | IL10, LRPAP1, RELN, LRP1, IL1B, VLDLR, A2M, CLU, LRP8, SORL1 |
| GO:0051091~positive regulation of sequence-specific DNA binding transcription factor activity | 5 | 1.64E-05 | IL10, RELN, IL1B, CLU, LRP8 |
| GO:0032501~multicellular organismal process | 13 | 2.87E-05 | IL10, LRPAP1, LRP1, LRP2, VLDLR, CLU, LRP8, SORL1, RELN, ATP7B, IL1B, A2M, LDLR |
| GO:0008202~steroid metabolic process | 5 | 3.19E-05 | IL1B, LRP2, VLDLR, LDLR, SORL1 |
| GO:0051128~regulation of cellular component organization | 9 | 3.90E-05 | IL10, LRPAP1, RELN, LRP1, IL1B, VLDLR, CLU, LRP8, SORL1 |
| GO:0051347~positive regulation of transferase activity | 6 | 5.02E-05 | RELN, IL1B, VLDLR, CLU, LRP8, SORL1 |
| GO:0044093~positive regulation of molecular function | 8 | 9.02E-05 | IL10, RELN, IL1B, VLDLR, A2M, CLU, LRP8, SORL1 |
| GO:0045859~regulation of protein kinase activity | 6 | 9.40E-05 | RELN, IL1B, VLDLR, CLU, LRP8, SORL1 |

**Table S13. The Drugs related to each group of genes.**

| **Number of Clusters** | **Number of Drugs** | **Name of Drugs** |
| --- | --- | --- |
| 1 | 712 | MOMETASONE FUROATE, MORICIZINE HYDROCHLORIDE, HYDROCORTISONE SODIUM SUCCINATE, CEFPODOXIME PROXETIL, BENAZEPRIL HYDROCHLORIDE, SALMETEROL , NORTRIPTYLINE HYDROCHLORIDE, DIGITOXIN , BETAMETHASONE , TIBOLONE , PREDNISOLONE , DYDROGESTERONE , TESTOLACTONE , PRIMIDONE , HYDROCORTISONE BUTYRATE, ESTRIOL , DESOXYCORTICOSTERONE PIVALATE, LANATOSIDE C, CHLORHEXIDINE HYDROCHLORIDE, UNOPROSTONE ISOPROPYL, GENTIAN VIOLET, FORMESTANE , DEQUALINIUM CHLORIDE, FLUOCINOLONE ACETONIDE, DOFETILIDE , THONZONIUM BROMIDE, CANDESARTAN CILEXETIL, HYDROCORTISONE , OXIGLUTATIONE , EFAVIRENZ , NORETHINDRONE , SPIRONOLACTONE , NELFINAVIR MESYLATE, DEFERASIROX , MEDRYSONE , ETHACRYNIC ACID, MITOXANTRONE HYDROCHLORIDE, SAQUINAVIR MESYLATE, HALOBETASOL PROPIONATE, PROBENECID , SIROLIMUS , DIFLUPREDNATE , RIFAPENTINE , DEFLAZACORT , NIFEDIPINE , HEXACHLOROPHENE , NANDROLONE DECANOATE, ZOLPIDEM , VALRUBICIN , DOMIPHEN BROMIDE, ESTRONE , DESLANOSIDE , AMCINONIDE , AMLODIPINE BESYLATE, COLCHICINE , MELOXICAM , FLUOXYMESTERONE , CAFFEINE , BORTEZOMIB , FENTANYL , DAROLUTAMIDE , DISULFIRAM , NICARDIPINE , AMDINOCILLIN , CHOLECALCIFEROL , ETONOGESTREL , ENZALUTAMIDE , FINASTERIDE , CANRENOATE POTASSIUM, HOMATROPINE HYDROBROMIDE, PIMOZIDE , TESTOSTERONE , TIZANIDINE , LACTIC ACID, OXYMETHOLONE , ZIPRASIDONE , PROMETHAZINE HYDROCHLORIDE, DUTASTERIDE , PRAZOSIN , DACTINOMYCIN , PODOFILOX , PHENELZINE SULFATE, CHLOROTRIANISENE , CLOMIPRAMINE HYDROCHLORIDE, CODEINE , PRAZEPAM , DOCUSATE SODIUM, NILUTAMIDE , LEVONORGESTREL , MESTRANOL , DIFLORASONE DIACETATE, DESERPIDINE , HALOPROGIN , DAUNORUBICIN HYDROCHLORIDE, ERLOTINIB , THIOTEPA , TRIAMCINOLONE DIACETATE, EVEROLIMUS , DROMOSTANOLONE PROPIONATE, PREDNISOLONE ACETATE, TOREMIFENE CITRATE, AMLEXANOX , DIETHYLCARBAMAZINE CITRATE, NORETHINDRONE ACETATE, FLUOCINONIDE , ALCLOMETASONE DIPROPIONATE, LOPERAMIDE , CHENODIOL , TACROLIMUS , CICLOPIROX OLAMINE, FEBUXOSTAT , CARBARIL , PACLITAXEL , ESTRAMUSTINE PHOSPHATE, TRIAMCINOLONE , ESTRADIOL BENZOATE, ATRACURIUM BESYLATE, VINBLASTINE SULFATE, IDARUBICIN HYDROCHLORIDE, FLUTAMIDE , MEPREDNISONE , FLURANDRENOLIDE , GRISEOFULVIN , FLUOXETINE HYDROCHLORIDE, NEFAZODONE HYDROCHLORIDE, CALCIPOTRIENE , ZAFIRLUKAST , METILDIGOXIN , DAUNORUBICIN , ESTRADIOL , PENTAMIDINE ISETHIONATE, HEXYLRESORCINOL , MITOBRONITOL , QUININE SULFATE, SORAFENIB TOSYLATE, CALCIFEDIOL , TAMOXIFEN CITRATE, PAROXETINE , ESTRADIOL VALERATE, PROGESTERONE , HALCINONIDE , PREDNICARBATE , OUABAIN , EXEMESTANE , THIOGUANINE , DEXAMETHASONE , MEPHOBARBITAL , CYCLANDELATE , MINOCYCLINE HYDROCHLORIDE, SOTALOL , BICALUTAMIDE , MENADIONE , CALCITRIOL , VINCRISTINE , VINORELBINE TARTRATE, TOLVAPTAN , NORGESTIMATE , BROMOCRIPTINE , LOTEPREDNOL ETABONATE, OXAZEPAM , TAZOBACTAM SODIUM, TRICLOCARBAN , PITAVASTATIN CALCIUM, CHLORPROMAZINE HYDROCHLORIDE, CROMOLYN SODIUM, TIOCONAZOLE , HYDROCORTISONE PROBUTATE, DESOXIMETASONE , MEDROXYPROGESTERONE ACETATE, METHYLPREDNISOLONE , LORACARBEF , NORETHYNODREL , ESTRADIOL CYPIONATE, ERGOCALCIFEROL , ACETOPHENAZINE , PARAMETHASONE ACETATE, HALOPERIDOL , PYRVINIUM PAMOATE, CEFONICID SODIUM, TESTOSTERONE PROPIONATE, TRILOSTANE , GEMCITABINE HYDROCHLORIDE, MEZLOCILLIN , ETHYLESTRENOL , CHLORDIAZEPOXIDE , DRONEDARONE , BUPRENORPHINE HYDROCHLORIDE, ESTRAMUSTINE PHOSPHATE SODIUM, ISOTRETINOIN , CARFILZOMIB , PIRETANIDE , MAPROTILINE HYDROCHLORIDE, DIAZEPAM , PERICIAZINE , MYCOPHENOLIC ACID, OCTOCRYLENE , ULIPRISTAL , MOCLOBEMIDE , METHYLTESTOSTERONE , RIFAMYCIN SODIUM, TRICLOSAN , AMIODARONE HYDROCHLORIDE, RETINOL , ETHYNODIOL DIACETATE, APOMORPHINE , ATROPINE , TESTOSTERONE ENANTHATE, PAPAVERINE HYDROCHLORIDE, CICLESONIDE , AMOROLFINE , ATALUREN , TESTOSTERONE CYPIONATE, PYRIMETHAMINE , METHYLENE BLUE, DIENOGEST , REPAGLINIDE , DULOXETINE HYDROCHLORIDE, LERCANIDIPINE HYDROCHLORIDE, APALUTAMIDE , MYCOPHENOLATE MOFETIL, HYDROCORTISONE SODIUM PHOSPHATE, BECLOMETHASONE DIPROPIONATE, MIFEPRISTONE , TELITHROMYCIN , CLOCORTOLONE PIVALATE, SULFISOXAZOLE , NORELGESTROMIN , MECHLORETHAMINE HYDROCHLORIDE, IOPAMIDOL , MITOTANE , ECONAZOLE NITRATE, MEGESTROL ACETATE, RIFAMPIN , DANAZOL , TOPOTECAN HYDROCHLORIDE, NISOLDIPINE , SERTRALINE HYDROCHLORIDE, NORGESTREL , IDEBENONE , TANNIC ACID, RALOXIFENE HYDROCHLORIDE, MELPHALAN , BIFONAZOLE , ROCURONIUM BROMIDE, MIDAZOLAM HYDROCHLORIDE, THIMEROSAL , PRASTERONE , CETRIMIDE , FLUFENAMIC ACID, BROMOCRIPTINE MESYLATE, SIMVASTATIN , POLIDOCANOL , DASATINIB , OXYBENZONE , TRIAMCINOLONE ACETONIDE, LULICONAZOLE , CARBAMAZEPINE , DIGOXIN , CLOTRIMAZOLE , FLUOROMETHOLONE , RABEPRAZOLE SODIUM, CYPROTERONE ACETATE, DESONIDE , GESTRINONE , ETHINYL ESTRADIOL, PAZOPANIB , FLUPHENAZINE , FENOFIBRATE , LOFEPRAMINE HYDROCHLORIDE, CHLORAMPHENICOL PALMITATE, ERGONOVINE , FLUOXETINE , FLUPREDNISOLONE , DESOXYCORTICOSTERONE ACETATE, METHYLPREDNISOLONE ACETATE, NICARDIPINE HYDROCHLORIDE, HYDROCORTISONE VALERATE, BETAMETHASONE SODIUM PHOSPHATE, OXANDROLONE , METHYLPREDNISOLONE SODIUM SUCCINATE, PYRIDOSTIGMINE BROMIDE, AZACITIDINE , CHLOROXINE , DROSPIRENONE , STANOZOLOL , DIDANOSINE , DOXORUBICIN HYDROCHLORIDE, KETOCONAZOLE , FLUSPIRILENE , MITOMYCIN , CHLORQUINALDOL , DIPHENOXYLATE HYDROCHLORIDE, DYCLONINE HYDROCHLORIDE, PREDNISONE , TRIAMTERENE , BETAMETHASONE DIPROPIONATE, BISACODYL , NANDROLONE PHENPROPIONATE, ABIRATERONE , WARFARIN POTASSIUM, PROMAZINE HYDROCHLORIDE, TAMOXIFEN , CLOFARABINE , TESTOSTERONE UNDECANOATE, TAMSULOSIN HYDROCHLORIDE, FLUNISOLIDE , METFORMIN HYDROCHLORIDE, FLUCYTOSINE , CLOBETASOL PROPIONATE, INDINAVIR SULFATE, NITAZOXANIDE , BENORILATE , DESOGESTREL , BETAMETHASONE VALERATE, LORATADINE , PAROXETINE HYDROCHLORIDE, SIROLIMUS , IRINOTECAN , DASATINIB , AMOXAPINE , THIMEROSAL , ECONAZOLE NITRATE, TRIAMTERENE , NICLOSAMIDE , AZATHIOPRINE , RALOXIFENE , ENCORAFENIB , NITAZOXANIDE , CAPECITABINE , CETUXIMAB , TRAMETINIB , FLUPHENAZINE , METHYLPREDNISOLONE , CYTARABINE , DOXORUBICIN HYDROCHLORIDE, RITUXIMAB , CARBOPLATIN , DOCETAXEL , MITOXANTRONE , BORTEZOMIB , DAUNORUBICIN , AZACITIDINE , ENALAPRIL , FURAZOLIDONE , IBRUTINIB , VORINOSTAT , APOMORPHINE , MERCAPTOPURINE , FENOFIBRATE , CINNARIZINE , LOPERAMIDE , SERTRALINE , CHLORAMBUCIL , SULCONAZOLE NITRATE, DABRAFENIB , TEMOZOLOMIDE , PROCHLORPERAZINE EDISYLATE, PANITUMUMAB , TRIFLURIDINE , BENZALKONIUM CHLORIDE, HALOPERIDOL , PRAMLINTIDE , CLADRIBINE , MENADIONE , MITOXANTRONE HYDROCHLORIDE, LORLATINIB , CLOTRIMAZOLE , MECHLORETHAMINE HYDROCHLORIDE, SALMETEROL XINAFOATE, EPIRUBICIN , BEVACIZUMAB , METHYLENE BLUE, CISPLATIN , METHYLDOPA , METHIMAZOLE , ANISINDIONE , ABEMACICLIB , SERTRALINE HYDROCHLORIDE, PROPYLTHIOURACIL , TRIFLUOPERAZINE , WARFARIN , MELPHALAN , IFOSFAMIDE , GRANISETRON , CLOFIBRATE , PACLITAXEL , CHLOROXINE , HYDRALAZINE HYDROCHLORIDE, ERLOTINIB , CLOMIPRAMINE , CLEMASTINE , ALPELISIB , CYCLOPHOSPHAMIDE , METHOTREXATE , TAMOXIFEN CITRATE, TAMOXIFEN , OLAPARIB , GEMCITABINE , HEXACHLOROPHENE , VENETOCLAX , DUVELISIB , VEMURAFENIB , DOXORUBICIN , PERPHENAZINE , ETHOPROPAZINE HYDROCHLORIDE, DAUNORUBICIN HYDROCHLORIDE, PROCHLORPERAZINE , FLUOROURACIL , TRICLOCARBAN , TRIFLUPROMAZINE , TOPOTECAN , NORTRIPTYLINE , DOPAMINE , PROGESTERONE , RIBAVIRIN , MAPROTILINE , CHLORPROMAZINE , CRIZOTINIB , ETOPOSIDE , DECITABINE , MITOMYCIN , OXALIPLATIN , PEMBROLIZUMAB , PIMOZIDE , PAZOPANIB , ETONOGESTREL , TIBOLONE , EXEMESTANE , DESOGESTREL , TOREMIFENE , CHOLECALCIFEROL , LAPATINIB , ESTROGENS, CONJUGATED, GENTIAN VIOLET, DACTINOMYCIN , ESTROGENS, ESTERIFIED, PROGESTERONE , BAZEDOXIFENE , QUINESTROL , POLYESTRADIOL PHOSPHATE, OXYBENZONE , PHENAZOPYRIDINE HYDROCHLORIDE, METHOXSALEN , TESTOSTERONE PROPIONATE, HEXACHLOROPHENE , TAMOXIFEN CITRATE, STANOZOLOL , IXABEPILONE , METHYLTESTOSTERONE , DOXYCYCLINE , ESTRONE , DANAZOL , DIETHYLSTILBESTROL DIPHOSPHATE, ABEMACICLIB , PERTUZUMAB , GOSERELIN , PERMETHRIN , TRASTUZUMAB , METHYLDOPA , NERATINIB , EVEROLIMUS , ANASTROZOLE , ESTROPIPATE , OXYTETRACYCLINE HYDROCHLORIDE, IODINE , LETROZOLE , ALLYLESTRENOL , NORELGESTROMIN , TRILOSTANE , PHENOLPHTHALEIN , ETHYNODIOL DIACETATE, LEVONORGESTREL , CHLOROTRIANISENE , MESTRANOL , TRIAMTERENE , LINDANE , DIENESTROL , ESTRADIOL VALERATE, PRASTERONE , CAMPHOR , FLUOXYMESTERONE , DOMIPHEN BROMIDE, ESTRADIOL CYPIONATE, ESTRADIOL ACETATE, TOREMIFENE CITRATE, GESTRINONE , ETHINYL ESTRADIOL, CISPLATIN , TAMOXIFEN , NORETHYNODREL , RETINOL , RIBOCICLIB , NORGESTREL , OXYMETHOLONE , CLOMIPHENE CITRATE, MITOTANE , NALOXONE , MELATONIN , DOXORUBICIN HYDROCHLORIDE, MEDROXYPROGESTERONE ACETATE, OLAPARIB , SYNTHETIC CONJUGATED ESTROGENS,, DOBUTAMINE , TESTOSTERONE , BAZEDOXIFENE ACETATE, DOCETAXEL , FULVESTRANT , ERIBULIN , LEFLUNOMIDE , ALPELISIB , RALOXIFENE , ESTRADIOL , CLOMIPHENE , OCTOCRYLENE , NORGESTIMATE , BORIC ACID, DAUNORUBICIN HYDROCHLORIDE, OSPEMIFENE , ESTROGENS, CONJUGATED SYNTHETIC, DIENOGEST , ESTRIOL , PYROGALLOL , PALBOCICLIB , LASOFOXIFENE , CHLOROTRIANISENE , TOREMIFENE , ETHINYL ESTRADIOL, PROGESTERONE , DIETHYLSTILBESTROL DIPHOSPHATE, PALBOCICLIB , OXYBENZONE , RALOXIFENE , EXEMESTANE , ESTRONE , TAMOXIFEN , PRASTERONE , LETROZOLE , IXABEPILONE , PERTUZUMAB , ABEMACICLIB , SYNTHETIC CONJUGATED ESTROGENS,, ESTROGENS, ESTERIFIED, EVEROLIMUS , FULVESTRANT , BAZEDOXIFENE , GOSERELIN , OSPEMIFENE , DOCETAXEL , TRILOSTANE , ESTROPIPATE , NERATINIB , TRASTUZUMAB , OCTOCRYLENE , GEMCITABINE , ALPELISIB , ESTRIOL , QUINESTROL , RIBOCICLIB , ESTRAMUSTINE PHOSPHATE SODIUM, ERIBULIN , ANASTROZOLE , LAPATINIB , OLAPARIB , ESTRADIOL , RALOXIFENE HYDROCHLORIDE, LASOFOXIFENE , TOREMIFENE CITRATE, BAZEDOXIFENE ACETATE, ESTROGENS, CONJUGATED SYNTHETIC, ESTROGENS, CONJUGATED, AMIODARONE , BALSALAZIDE , LEVOTHYROXINE , SPIRONOLACTONE , INDOMETHACIN , SULFASALAZINE , PIOGLITAZONE , PHENOLPHTHALEIN , MESALAMINE , BEXAROTENE , GLIPIZIDE , REPAGLINIDE , IBUPROFEN , PIOGLITAZONE HYDROCHLORIDE, BEZAFIBRATE , OLANZAPINE , RALOXIFENE HYDROCHLORIDE, CLOFAZIMINE , METHOTREXATE , GENTIAN VIOLET, ZAFIRLUKAST , FISH OIL TRIGLYCERIDES, GLYBURIDE , BALSALAZIDE DISODIUM, DEXIBUPROFEN , TELMISARTAN , BENZBROMARONE , ESTRADIOL , ZOLEDRONIC ACID, DICLOFENAC , TREPROSTINIL , NATEGLINIDE , FENOPROFEN , ASPIRIN , MYCOPHENOLIC ACID, FENOFIBRIC ACID, OLSALAZINE SODIUM, CANNABIDIOL , TRICLOSAN , NOREPINEPHRINE , INAMRINONE , ISOPROTERENOL , CALCIUM , ALITRETINOIN , RABEPRAZOLE SODIUM, TAMOXIFEN , MENADIONE , CEFOTAXIME , HEXACHLOROPHENE , METHOTREXATE , IRINOTECAN , ZAFIRLUKAST , ECONAZOLE NITRATE, ISOTRETINOIN , PANTOPRAZOLE , GAMOLENIC ACID, IDEBENONE , RABEPRAZOLE , DOCUSATE , ETHACRYNIC ACID, DOXERCALCIFEROL , MEDROXYPROGESTERONE ACETATE, THIMEROSAL , GENTIAN VIOLET, DACTINOMYCIN , MITOXANTRONE HYDROCHLORIDE, MICONAZOLE , EDARAVONE , MESALAMINE , OMEPRAZOLE , AMOXAPINE , DIHYDROTACHYSTEROL , CARBARIL , LOVASTATIN , BENZBROMARONE , CARBOPLATIN , EPINEPHRINE , DEXAMETHASONE , CYSTEINE , HYDROCORTISONE , TACALCITOL , BRIMONIDINE , MIDAZOLAM , BENSERAZIDE , NIFEDIPINE , IODOFORM , ACITRETIN , CALCIFEDIOL , TESTOSTERONE , ALENDRONIC ACID, CEFACLOR , CEFPIROME , PYRANTEL PAMOATE, CALCIPOTRIENE , DYCLONINE , DISULFIRAM , KETOCONAZOLE , NISOLDIPINE , OXYTETRACYCLINE HYDROCHLORIDE, URACIL MUSTARD, TOLFENAMIC ACID, CAFFEINE , MERSALYL , CHOLESTEROL , ALFACALCIDOL , NITAZOXANIDE , METHYLERGONOVINE , BROMOCRIPTINE , THIOTEPA , BOCEPREVIR , PARICALCITOL , CYCLOSPORINE , METHYSERGIDE , CHOLECALCIFEROL , DESERPIDINE , LOFEPRAMINE , WARFARIN , CANDESARTAN CILEXETIL, APOMORPHINE , LANSOPRAZOLE , RITONAVIR , PYRITHIONE , RIBAVIRIN , DAUNORUBICIN HYDROCHLORIDE, ISOETHARINE , OXCARBAZEPINE , CALCITRIOL , RALOXIFENE , ERGOCALCIFEROL , ACRIFLAVINE , CYSTEAMINE HYDROCHLORIDE, HALOPROGIN , DYCLONINE HYDROCHLORIDE, SULFINPYRAZONE , CEFONICID , TRETINOIN , PYROGALLOL , DEFERASIROX , CALCIFEDIOL , MALATHION , TRIMETREXATE , ESTROPIPATE |
| 3 | 60 | DICLOFENAC SODIUM, ARTEMISININ, DEXIBUPROFEN, BIFONAZOLE, FORMESTANE, FLUCONAZOLE, NAPROXEN SODIUM, CHENODIOL, METHOTREXATE, RILUZOLE, ESTRONE, SIMVASTATIN, TESTOSTERONE PROPIONATE, FLURBIPROFEN, PROGESTERONE, IBUPROFEN, PRASTERONE, RITONAVIR, METHOTREXATE, MIFAMURTIDE, TACROLIMUS, SAQUINAVIR, ALCOHOL, NELFINAVIR, NALOXONE, INFLIXIMAB, CYCLOBENZAPRINE, PRAVASTATIN, TESTOSTERONE, PIRFENIDONE, TAMOXIFEN, IRINOTECAN, AMIFOSTINE, TERAZOSIN, ETOPOSIDE, NICOTINE, TRIAMCINOLONE, STREPTOZOCIN, RITUXIMAB, HYDROCORTISONE, PIOGLITAZONE, TOREMIFENE, HYALURONIDASE, VERAPAMIL, ATENOLOL, CLADRIBINE, DOXORUBICIN, RAMIPRIL, ASPIRIN, MELATONIN, ALUMINUM ACETATE, DAUNORUBICIN, DIMERCAPROL, HYDROXYCHLOROQUINE, TROMETHAMINE, GENTIAN VIOLET, PROPOFOL, DEFEROXAMINE, COPPER, METHYLDOPA |
| 6 | 208 | DEXMETHYLPHENIDATE HYDROCHLORIDE, AMPHETAMINE SULFATE, VENLAFAXINE, PSEUDOEPHEDRINE, COCAINE, AMOXAPINE, DEXTROAMPHETAMINE ADIPATE, DISULFIRAM, PHENELZINE, LEVODOPA, DIETHYLPROPION HYDROCHLORIDE, HALOPERIDOL, ESCITALOPRAM, LEVOMILNACIPRAN, NICOTINE, BUPROPION HYDROCHLORIDE, DEXTROAMPHETAMINE SULFATE, DEXTROAMPHETAMINE, SOLRIAMFETOL, DIPHENYLPYRALINE, LOXAPINE, ACETYLCYSTEINE, MIANSERIN, SERTRALINE, LISDEXAMFETAMINE DIMESYLATE, CHLORPHENIRAMINE, MAZINDOL, MEPERIDINE, CLOMIPRAMINE, NEFAZODONE, DULOXETINE, BENZPHETAMINE, AMPHETAMINE ADIPATE, METHYLPHENIDATE, AMPHETAMINE ASPARTATE, METHYLPHENIDATE HYDROCHLORIDE, PHENMETRAZINE HYDROCHLORIDE, AMPHETAMINE, DEXMETHYLPHENIDATE, CHLOROPROCAINE, BENZTROPINE, ATOMOXETINE, ARIPIPRAZOLE, DESVENLAFAXINE, IMIPRAMINE, DOPAMINE, MODAFINIL, PROCAINE, TRIMIPRAMINE, BUPROPION HYDROBROMIDE, METYROSINE, CLOZAPINE, LISDEXAMFETAMINE, ARMODAFINIL, PHENMETRAZINE, MIRTAZAPINE, DIETHYLPROPION, PHENTERMINE, BUPROPION, TAPENTADOL, ZIPRASIDONE, RISPERIDONE, MAZINDOL, SERTRALINE, DOPAMINE, DAPOXETINE, MEPERIDINE, AMOXAPINE, LEVOMILNACIPRAN, TRAMADOL, PHENTERMINE, ALCOHOL, METHADONE, COCAINE, MIANSERIN, DESIPRAMINE, LOXAPINE, ATOMOXETINE, OLANZAPINE, LOFEPRAMINE, BUPRENORPHINE, NORTRIPTYLINE, VENLAFAXINE, FLUVOXAMINE, ZOTEPINE, SOLRIAMFETOL, PAROXETINE, MIRTAZAPINE, DEXMETHYLPHENIDATE, IMIPRAMINE, HALOPERIDOL, RIBAVIRIN, VORTIOXETINE, PSEUDOEPHEDRINE, ONDANSETRON, TRIMIPRAMINE, PROTRIPTYLINE, CLOZAPINE, BUPROPION, VERAPAMIL, ESCITALOPRAM, MILNACIPRAN, METHYLPHENIDATE, ARIPIPRAZOLE, CHLORPHENIRAMINE, PHENELZINE, CYCLOBENZAPRINE, CITALOPRAM, TRAZODONE, DOXEPIN, VILAZODONE, DOTHIEPIN, MORPHINE, DULOXETINE, FLUOXETINE, AMITRIPTYLINE, NEFAZODONE, DESVENLAFAXINE, CLOMIPRAMINE, QUETIAPINE, AMPHETAMINE, DEXTROMETHORPHAN, BUTRIPTYLINE, ETOPOSIDE, CLOMIPRAMINE, PACLITAXEL, CYCLOPHOSPHAMIDE, BLEOMYCIN, HYDROQUINONE, IRINOTECAN HYDROCHLORIDE, SELENOMETHIONINE, PYRIMETHAMINE, DOCETAXEL, CARBOCYSTEINE, DOXORUBICIN, CARBOPLATIN, OXALIPLATIN, OMEPRAZOLE, CLOZAPINE, EPIRUBICIN, RIFAMPIN, FLUOROURACIL, AZACITIDINE, DEXAMETHASONE, CISPLATIN, IFOSFAMIDE, DECITABINE, GLYCERIN, THIOTEPA, VERAPAMIL, MELPHALAN, PREDNISONE, BUSULFAN, ISONIAZID, CYTARABINE, LEUCOVORIN, DAUNORUBICIN, ALCOHOL, PYRAZINAMIDE, RISPERIDONE, OMEPRAZOLE, CARBOPLATIN, INFLIXIMAB, ATORVASTATIN, METHYLENE BLUE, CERTOLIZUMAB PEGOL, ISONIAZID, ETANERCEPT, SPIRONOLACTONE, BINIMETINIB, EPINEPHRINE, PSEUDOEPHEDRINE, GENTAMICIN, GOLIMUMAB, BUPIVACAINE, MIDAZOLAM, GLIMEPIRIDE, SORAFENIB, DIDANOSINE, STAVUDINE, INAMRINONE, APREMILAST, LENALIDOMIDE, GLUCOSAMINE, CLENBUTEROL, RABEPRAZOLE, LACTULOSE, THALIDOMIDE, PENTOXIFYLLINE, ALTEPLASE, CYCLOSPORINE, INSULIN, ADALIMUMAB, GEMCITABINE, DIGOXIN, POMALIDOMIDE, MEROPENEM, ETHAMBUTOL, HYDROXYCHLOROQUINE, METHIMAZOLE, PROPYLTHIOURACIL, RIFAMPIN, MILTEFOSINE, AMPHOTERICIN B, PYRIDOXINE, CEFOTAXIME, CARBAMAZEPINE, CHLOROQUINE |
| 7 | 114 | AMITRIPTYLINE, METHADONE, FOSTAMATINIB, SORAFENIB, CLOZAPINE, PILOCARPINE, CORTICOTROPIN, ESKETAMINE, DOXORUBICIN, LAROTRECTINIB, CISPLATIN, PAZOPANIB HYDROCHLORIDE, ACALABRUTINIB, PONATINIB, NINTEDANIB, PEXIDARTINIB, PAZOPANIB, DASATINIB, FOSTAMATINIB, GEFITINIB, IMATINIB, IBRUTINIB, VANDETANIB, CYCLOPHOSPHAMIDE, FOSTAMATINIB, CRIZOTINIB, PILOCARPINE, TAMOXIFEN, METHOTREXATE, IMATINIB, SORAFENIB, CENEGERMIN, ACETYLCYSTEINE, DEXAMETHASONE, REGORAFENIB, PHENYLEPHRINE, ASPIRIN, LAROTRECTINIB, AMITRIPTYLINE, STREPTOZOCIN, GILTERITINIB, PRASUGREL, PYRIDOXINE, INSULIN, VINCRISTINE, COLCHICINE, CAPSAICIN, NALOXONE, CYCLOPHOSPHAMIDE, PHENYTOIN, NIMODIPINE, ALTEPLASE, LIOTHYRONINE SODIUM, EPOETIN ALFA, PILOCARPINE, RISPERIDONE, NICOTINE, HALOPERIDOL, LIOTHYRONINE SODIUM, NALOXONE, DOXORUBICIN, BACLOFEN, OLANZAPINE, IMIPRAMINE, PAROXETINE, PROPRANOLOL, VINCRISTINE, CITALOPRAM, INSULIN, TRAMADOL, LIDOCAINE, NICARDIPINE, MIFEPRISTONE, SUMATRIPTAN, COLCHICINE, GLUCAGON, GEMFIBROZIL, DONEPEZIL, PROPYLTHIOURACIL, NYSTATIN, DIACETYLMORPHINE, VERTEPORFIN, TOPOTECAN, METHADONE, PILOCARPINE, VASOPRESSIN, ESCITALOPRAM, VENLAFAXINE, CORTICOTROPIN, INDOMETHACIN, NICOTINE, NORTRIPTYLINE, LEVODOPA, BORTEZOMIB, THYROGLOBULIN, EPOETIN BETA, SODIUM CHLORIDE, CETUXIMAB, FLUOROURACIL, SIMVASTATIN, GENTAMICIN, ALTEPLASE, DESIPRAMINE, METHYLPREDNISOLONE, FLURBIPROFEN, FLUOXETINE, ESKETAMINE, CHLORPROMAZINE, METYROSINE, CAPSAICIN, PACLITAXEL, DRONABINOL, COPPER, DIDANOSINE |
| 8 | 63 | CLARITHROMYCIN, EFAVIRENZ, ACYCLOVIR, RITUXIMAB, AMOXICILLIN, TACROLIMUS, TRETINOIN, ZIDOVUDINE, RABEPRAZOLE, CYCLOSPORINE, MESALAMINE, SIROLIMUS, CHOLESTYRAMINE, ATORVASTATIN, ACETYLCYSTEINE, EVOLOCUMAB SODIUM, PORFIMER, CORTICOTROPIN ALFA, EPOETIN, VERAPAMIL, MIPOMERSEN, GEMFIBROZIL, RIBAVIRIN, LOVASTATIN, ATENOLOL, ALIROCUMAB, GLUCAGON, HALOPERIDOL, PRAVASTATIN, LOMITAPIDE, RETINOL, CEFACLOR, HYDROCORTISONE, USTEKINUMAB, ACITRETIN, PRAVASTATIN, CANAKINUMAB, GLUCOSAMINE, THYROGLOBULIN ACID, RISEDRONIC, ERYTHROMYCIN, RILONACEPT, VERAPAMIL, RABEPRAZOLE, PENTOXIFYLLINE NITRATE, GALLIUM ACID, TILUDRONIC, RALOXIFENE, DIACEREIN, PENTAMIDINE, MELATONIN, OFLOXACIN, NICARDIPINE, HYDROQUINONE, OMEPRAZOLE, LANSOPRAZOLE, ALTEPLASE, MORPHINE, DONEPEZIL, INFLIXIMAB, MINOCYCLINE, ASPIRIN, BINIMETINIB |
